# Revealing human sensitivity to a latent temporal structure of changes

**DOI:** 10.3389/fnbeh.2022.962494

**Published:** 2022-10-17

**Authors:** Dimitrije Marković, Andrea M. F. Reiter, Stefan J. Kiebel

**Affiliations:** ^1^Department of Psychology, Technische Universität Dresden, Dresden, Germany; ^2^Department of Child and Adolescence Psychiatry, Psychosomatics and Psychotherapy, Centre of Mental Health, University Hospital Würzburg, Würzburg, Germany; ^3^German Center of Prevention Research on Mental Health, Julius-Maximilians Universität Würzburg, Würzburg, Germany; ^4^Centre for Tactile Internet with Human-in-the-Loop (CeTI), Technische Universität Dresden, Dresden, Germany

**Keywords:** decision making, temporal structure, Bayesian inference, active inference, reversal learning

## Abstract

Precisely timed behavior and accurate time perception plays a critical role in our everyday lives, as our wellbeing and even survival can depend on well-timed decisions. Although the temporal structure of the world around us is essential for human decision making, we know surprisingly little about how representation of temporal structure of our everyday environment impacts decision making. How does the representation of temporal structure affect our ability to generate well-timed decisions? Here we address this question by using a well-established dynamic probabilistic learning task. Using computational modeling, we found that human subjects' beliefs about temporal structure are reflected in their choices to either exploit their current knowledge or to explore novel options. The model-based analysis illustrates a large within-group and within-subject heterogeneity. To explain these results, we propose a normative model for how temporal structure is used in decision making, based on the semi-Markov formalism in the active inference framework. We discuss potential key applications of the presented approach to the fields of cognitive phenotyping and computational psychiatry.

## 1. Introduction

The passage of time is a fundamental aspect of human experience. Our behavior is tightly coupled to our estimate of the elapsed time and the expectations about the time remaining to fulfill short or long-term goals. We are highly sensitive to the temporal structure of our everyday environment and capable of forming precise beliefs about the duration of various events (e.g., a theater play, traffic lights, waiting in a queue). In practice, temporal structure is typically latent (e.g., not reflected in external clocks) and we seem to rely on an internalized timing mechanism, such as various implicit clocking mechanisms (Buhusi and Meck, 2005). This enables us to provide temporal context and an order to events, and to form beliefs about the underlying temporal structure (Eichenbaum, 2014). It has been proposed that these temporal beliefs are used to make predictions and to adapt our behavior successfully to ever-changing conditions (Griffiths and Tenenbaum, 2011). Therefore, understanding how we learn and represent the temporal structure of our every day environment (Kiebel et al., 2008) and use these representations for making decisions (Marković et al., 2019) is essential for understanding human adaptive behavior (Purcell and Kiani, 2016).

Neuronal and behavioral mechanisms of time perception have been studied in humans and animals, traditionally using interval timing tasks (Meck, 1996; Eagleman, 2008). The key insights of these experiments are that humans and animals integrate the experience of between event duration, in a given context, to form beliefs about possible future duration they might experience. They use these beliefs when estimating or reproducing a newly experienced interval (Jazayeri and Shadlen, 2010); in line with a Bayesian account of decision-making (Shi et al., 2013). However, it is still an open question how we integrate time perception and beliefs about durations into everyday decision making. Recently, distinct but interlinked research fields have illustrated the importance of temporal representations for cognition and decision making in sequential and dynamic tasks (McGuire and Kable, 2012; Eichenbaum, 2014; Vilà-Balló et al., 2017; Nobre and Van Ede, 2018). The sequential neuronal activity in the hippocampus has been suggested to represent elapsed time (Friston and Buzsáki, 2016; Buzsáki and Llinás, 2017; Eichenbaum, 2017), which have led to the postulate of time cells in the hippocampus (Itskov et al., 2011; Eichenbaum, 2014; MacDonald et al., 2014) critical for memory and decision-making. For example, in the research on temporal aspects of attention it has been demonstrated that temporal expectations guide allocation of attentional resources in time (Nobre and Van Ede, 2018). Similarly, inter-temporal choices or one's willingness to wait for higher reward is strongly influenced by temporal expectations (McGuire and Kable, 2012).

Motivated by the rich literature on temporal representations in the brain, here we focus on the question of how humans form complex temporal representation of their environment. We test how such temporal representations support decisions about whether to explore or to exploit in anticipation of a change in the environment. We introduce a novel computational model of behavior that describes learning of a latent temporal structure of a dynamic task environment in the context of sequential decision making. The computational model is applicable to any task that can be cast as a dynamic multi-armed bandit problem (Gupta et al., 2011) with semi-Markovian changes or switches in the underlying latent states (Janssen and Limnios, 1999). Here we specifically apply the model to describe learning in a sequential (probabilistic) reversal learning task (Costa et al., 2015; Reiter et al., 2016, 2017; Vilà-Balló et al., 2017). We do so by manipulating temporal contexts in this task: Subjects encountered semi-regular intervals between contingency reversals in one environment. Their behavior was contrasted with behavior in another environment where intervals between contingency reversals were irregular.

The proposed behavioral model was based on three components: (i) a set of templates representing possible latent temporal structure of reversals using an implicit representation of between reversal duration (Yu, 2015), (ii) the update of beliefs about states and temporal templates derived *via* approximate inference (Yu and Kobayashi, 2003; Parr et al., 2019), and (iii) the action selection, that is the planning process, cast as active inference (Friston et al., 2017; Markovic et al., 2021). Together these components allow us to define an efficient and approximate active learning and choice algorithm of latent temporal structures based on variational inference (Blei et al., 2017). Here we extend on our previous investigation of human behavior in temporally structured dynamic environments (Marković et al., 2019). In this work, we demonstrated that a computational model which infers a between-event duration, can be used to reveal subjects' beliefs about the latent temporal structure in a dynamic learning task. However, a question that has remained open is how humans acquire temporal structure in the first place. Understanding the learning of temporal structure is critical for revealing between-individual variability in temporal expectations and capturing the evolution of temporal representations within individuals. Critically, with the extended model we present here, we are indeed able to capture the learning of temporal representation and address the non-stationarity of subjects' temporal representation during the course of the experiment.

Our aim is to address the following questions: (i) Are subjects a priori biased toward expecting regular or irregular temporal structure? (ii) Are subjects able to learn latent temporal structure without explicit instructions? (iii) How does the quality of temporal representation impact their performance? Using simulations we can illustrate the interaction of accurate representation of temporal structure and behavior, mainly performance on the task and the engagement with exploratory behavior. Using model-based analysis, that is, by estimating the prior beliefs—under a semi-Markovian generative model—that best explain observed choice behavior, we demonstrate high diversity between subjects both in their prior beliefs about temporal structure, and their ability to adapt their beliefs to different latent temporal structure. Crucially, we link the quality of temporal representation to subjects' performance both in terms of group-level performance and within-subject variability of their performance during the task.

In what follows we will first briefly describe the experimental task, provide the overall summary of behavioral characteristics, introduce the behavioral model, and finally show results of the model-based analysis of behavior. The formal details of the approach are described in Section 4.

## 2. Results

**Figure 1:**
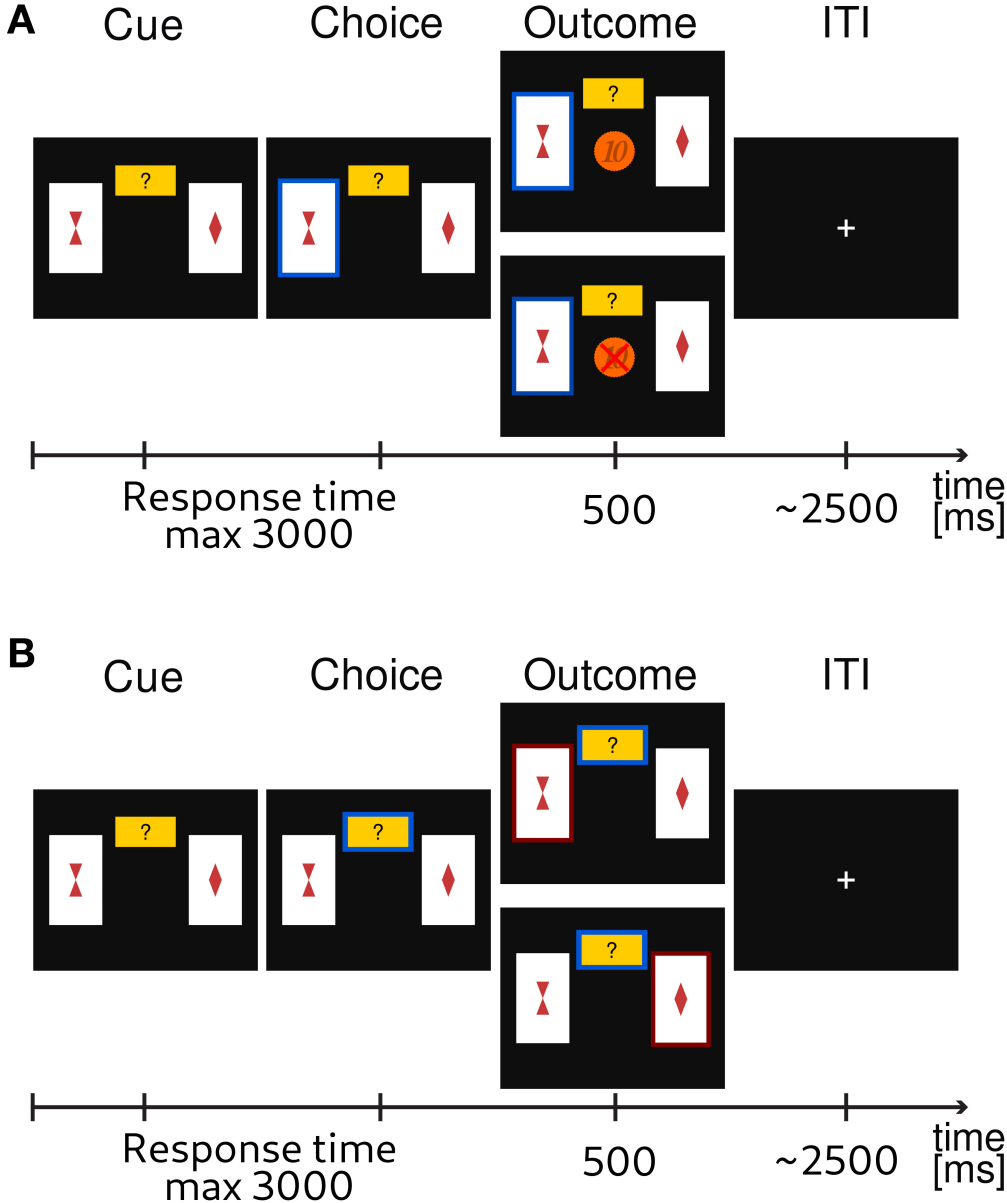
Exemplary trial sequence of the probabilistic reversal learning task. Subjects were instructed that one card always had a higher probability of a monetary reward. They were instructed to choose the card that they thought would lead to a monetary gain with higher probability, or, alternatively, choose to explore (small yellow rectangle with question mark). The latter would provide them with a correct information about what option would have had a higher probability of reward. **(A)** If participants had chosen one of the cards, the corresponding card was highlighted and feedback was displayed. The feedback consisted of either the visual display of a 10 Euro cents coin in the center of the screen for a gain outcome, or a crossed 10 Euro cents for a loss outcome. **(B)** If the participant had chosen the explore option, the card with the currently highest reward probability was highlighted (either the left or the right card).

A typical probabilistic reversal learning task asks subjects to make a binary choice between two options, e.g., A and B, where each option is associated with a probability of receiving a reward or punishment. For example, initially choosing A returns a reward with a high probability *p*_*H*_ = 0.8 and choosing B returns a reward with low probability *p*_*L*_ = 0.2. Importantly, after several trials the reward contingencies reverse, i.e., switch, such that choosing B returns the reward with high probability *p*_*H*_. However, subjects are not informed about the reversal and they have to infer that a change occurred from the feedback they receive in order to adapt their behavior. From the point of view of participants, a reversal can be difficult to detect as outcomes are probabilistic. This means that if someone observes a loss after a sequence of gains, e.g., when choosing the option A, this could be caused either by: (i) a true reversal, where now option B is rewarded with the probability *p*_*H*_ or (ii) by an unlucky outcome of an otherwise correct choice. To obtain a more direct information about the subjective uncertainty of participants about the correct choice (i.e., choosing the option with high reward probability, *p*_*H*_) on any given trial, we extended the standard design with an additional third exploratory option. This new option does not result in monetary gain or loss but provides information about the correct choice on a current trial. A high uncertainty about the best choice (current context) can be easily resolved by selecting the epistemic option. We will label all choices of the exploratory options as exploratory, and all other choices as exploitative (note that the outcomes of exploitative options also provide some information about the current context). A trial sequence of the experimental task is shown in Figure 1.

**Figure 2:**
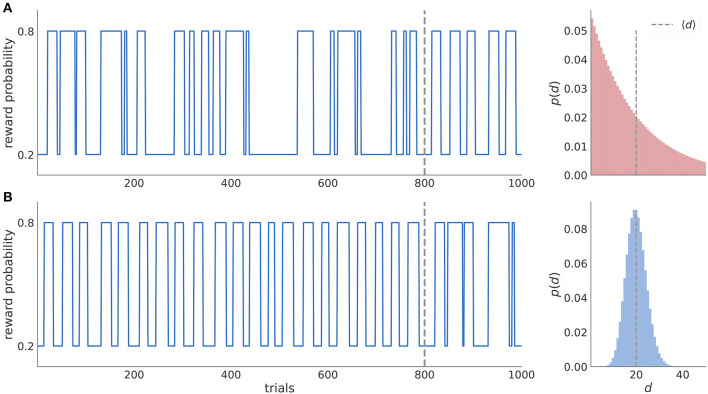
Time series of reward probabilities. **(A)** Condition with irregular reversals, and **(B)** condition with (semi-)regular reversals. The reward probability of the high-probability stimuli at any time step was set to *p*_*H*_ = 0.8 and the low-probability stimuli to *p*_*L*_ = 0.2. Dashed vertical lines shows the moment of change of the latent temporal structure: (i) from irregular to semi-regular statistics in the irregular condition, and (ii) from the semi-regular to irregular statistics in the regular condition. Figures on the right illustrate the generative distribution of the between-reversal intervals *d* for each condition. Note that the mean between reversal duration 〈*d*〉 is identical in both conditions.

To investigate subjects' ability to learn latent temporal structure we defined two experimental conditions (manipulated in a between-subject design), one with irregular reversals and another with regular reversals (see Figure 2). In the condition with irregular reversals, the moments of reversals are not predictable and between-reversal intervals are drawn from a geometric distribution (Figure 2A). In the condition with regular reversals, the moments of reversal are predictable, and they occur at semi-regular intervals, drawn from a negative binomial distribution (Figure 2B). Subject were randomly assigned to one of the two possible conditions, as illustrated in Figure 2. In the first condition, subjects experience irregular reversal statistics for 800 trials, after which the reversals occur at semi regular intervals for the last 200. In the second condition, subjects experience semi-regular reversal statistics for 800 trials, and then the irregular reversal statistics during the last 200 trials. Note that when changing the temporal statistics we copied the time series of reversals from the initial 200 trials of the different condition. The motivation for using parts of the trajectories from one condition in another condition comes from the process we use to define the moments of reversals in both conditions. We aimed to tailor both experimental conditions in a way that maximizes the behavioral differences between subjects entertaining different underlying beliefs about latent statistics of reversals. Such optimization results in improved model selection and parameter estimates as distinct latent beliefs result in more pronounced behavioral differences.

Therefore, we have generated a large number (10^5^) of trajectories of length *T* = 800 for each condition and kept the one for which we found the maximal performance difference between agents with a correct representation of latent reversal statistics and an agent with a representation from the opposite condition. As we kept only single trajectory of reversals for each condition, we have fixed the moments of reversal for each subject group (depending on the condition, reversals occur always on the same trials). Furthermore, the same choice by different subjects exposed to the same condition leads to the same outcome on any given trial (the outcome statistics were generated only once for each condition and trial, and then replayed to all subjects depending on their choices and the condition they were assigned to). Hence, we removed the noise in behavioral responses which would be induced by unique experiences of each subject in the experiment, were we to generate moments of reversal and response-outcomes on-the-fly for each subject.

### 2.1. Analysis of choice data

We will first describe the behavioral characteristics of the two groups of subjects exposed to the two different experimental conditions. The two behavioral measures of interests here are the *performance* (odds of being correct, i.e., odds of choosing the option with the higher reward probability) and *probing* (odds of exploring, i.e., odds of choosing the exploratory option). We describe all the behavioral measures in detail in Section 4.5.

**Figure 3:**
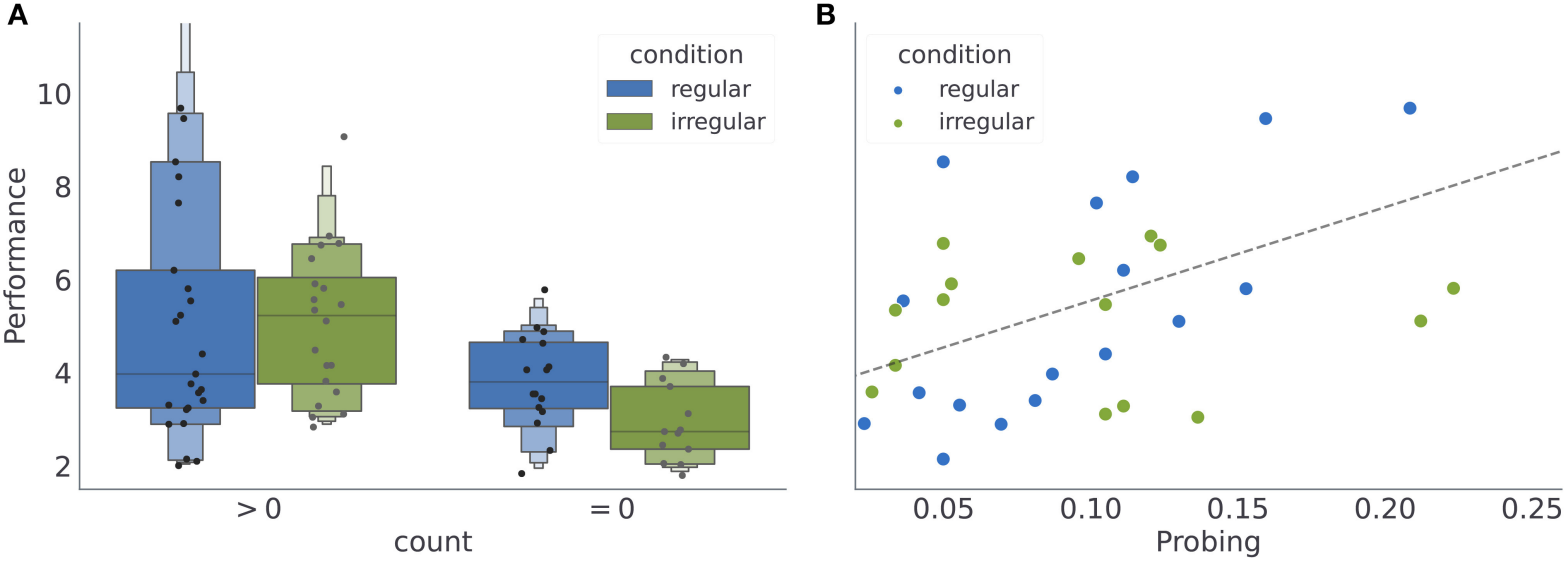
Averages of behavioral summary measures. **(A)** Distribution of the mean performance of subjects with low and high number of exploratory choices (see Section 4). **(B)** Dependence of the mean performance on the mean probing, where we excluded participants without exploratory choices (count = 0). Note that when computing mean performance and mean probing for each participants, we have excluded the first 400 (initial responses during which the subjects might have still been adjusting to the task) and last 200 (responses after the change in the reversal statistics) responses of each participant, see Section 4.7 for the motivation for the cutoff.

Subjects (*N* = 74) were pseudo-randomly assigned to one of the two experimental conditions, where *n*_*r*_ = 41 participants were assigned to the condition with regular reversals, and *n*_*i*_ = 33 to the condition with irregular reversals. Note that some subjects rarely engaged with exploratory option. Out of 50 subjects who where exposed to the variant of the experiment with exploratory option (24 subjects performed a standard version of the task without exploratory option, see Section 4.3 for more details), 5 subjects never engaged with the exploratory option. In Figure 3, we provide a summary of average behavioral measures for individual subjects. We do not find any significant performance differences between the two regularity conditions (see Figure 3A). However, for the subset of subjects which interacted with the exploratory option (45 subjects) we find that the performance is positively correlated with probing (Pearson correlation coefficient for all data points *r* = 0.6, with *p* < 10^−4^; for the regular condition *r* = 0.73, *p* < 0.0001, and for the irregular condition *r* = 0.52, *p* < 0.02; see Figure 3B). Interestingly, neither of the two behavioral measures (when plotted as a within subject average over the course of experiment), reveals obvious between-condition differences. However, when comparing the temporal profile of these measures over the course of experiment (see Supplementary Figure 1), one notices large variability both between subjects but also within a subject over the course of experiment; suggesting ongoing learning of the task structure. In what follows we will classify the heterogeneity of behavioral responses using a model-based analysis.

### 2.2. Behavioral model

The behavioral model will allow us to investigate the process of learning of the latent temporal structure in different experimental conditions, reveal subjects' preferences to engage with an exploratory option (collect information), and subjects' motivation to collect rewards. We achieve this by fitting free model parameters to behavioral responses of each subject (see Section 4 for more details). Our aim with the model based analysis is to quantify beliefs about temporal structure of reversals and understand how the belief updating influences subjects' behavior.

We conceptualized the behavioral model as an active inference agent (Friston et al., 2015, 2016) with hidden semi-Markov models (Yu, 2010), which are capable of representing and inferring latent temporal structure. In active inference, besides defining perception and learning as a Bayesian inference process, action selection is also cast as an inference problem aimed at minimizing the expected surprise about future outcomes, that is, the expected free energy (Smith et al., 2022; see also Equation 18). Through its dependence on the expected free energy, the action selection has an implicit dual imperative (see possible factorization of the expected free energy in Equation 18): The expected free energy combines intrinsic and extrinsic value of a choice, where intrinsic value corresponds to the expected information gain, and the extrinsic value to the expected reward of different choices. The implicit information gain or uncertainty reduction pertains to beliefs about the task's dynamical structure and choice-outcome mappings (e.g., Schwartenbeck et al., 2013; Kaplan and Friston, 2018). Therefore, selecting actions that minimize the expected free energy dissolves the exploration-exploitation trade-off, as every action is driven both by expected value and the expected information gain. This is a critical feature of active inference models which allows us to account for exploratory choices (see Figure 1).

We express the agent's generative model of task dynamics in terms of hidden semi-Markov models (HSMM) (Yu, 2015; Marković et al., 2019). The HSMM framework extends a standard hidden Markov model with an implicit (or explicit) representation of durations between consecutive state changes. HSMM have found numerous applications in the analysis of non-stationary time series in machine learning (Duong et al., 2005; Gales and Young, 2008), and in neuroimaging (Borst and Anderson, 2015; Shappell et al., 2019). HSMM have also been used in decision making for temporal structuring of behavioral policies (Bradtke and Duff, 1994) or in temporal difference learning as a model of dopamine activity when the timing between action and reward is varied between experimental trials (Daw et al., 2002).

**Figure 4:**
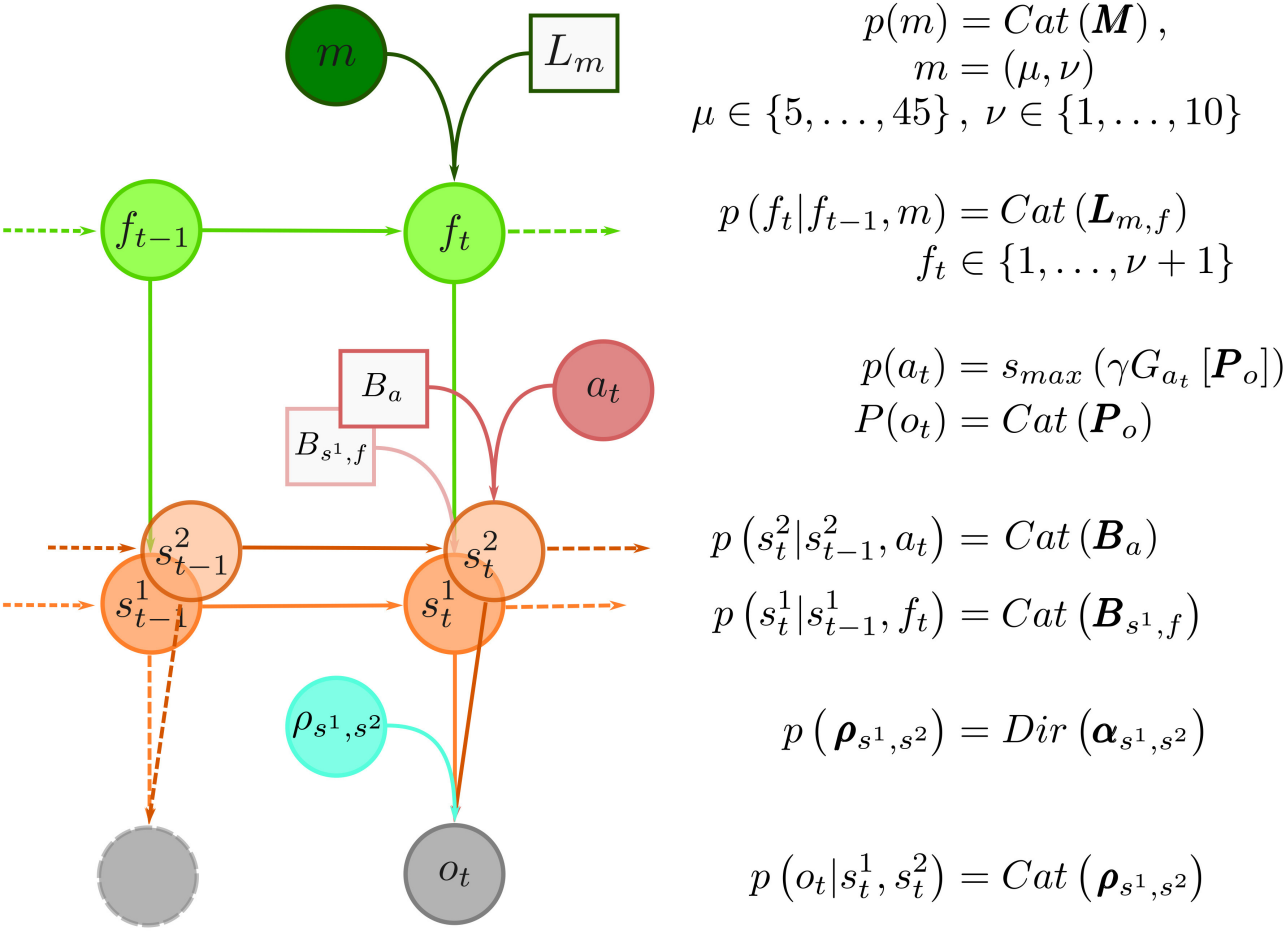
Graphical representation of the generative model and model summary. At the top of the hierarchy is the temporal template variable *m*. The total number of temporal templates is finite, e.g., *m* ∈ {1, …, *m*_*max*_}, and each template *m* provides an implicit representation of a prior probability distribution over between-reversal intervals *d*, parameterized with a pair *m* = (μ, ν), where μ expresses mean between reversal interval, and ν plays a role of a precision parameter, that defines regularity of between-reversal intervals. The implicit representation of temporal structure is encoded with probability transition matrices ***L***_*m,f*_ of latent phases *f*. The number of latent phases depends on precision ν. The reversal can occur only when the end phase is reached (*f* = ν + 1). Therefore, the phase variable *f* controls the transitions probability $B_{s^{1},f}$ between latent states of the task denoted as random variable *s*^1^ ∈ {1, 2}. At every trial *t* the subject makes a choice *a*_*t*_ hence decides on which option (*s*^2^) to select which results in an outcome *o*_*t*_. The choices are deterministic, meaning that the corresponding transition probability corresponds to identity matrix, that is, $p\left(s_{t}^{1}|s_{t-1}^{1},a_{t}\right)=p\left(s_{t}^{1}|a_{t}\right)=\delta_{s_{t}^{1},a_{t}}$, hence ***B***_*a*_ = *I*_3_. Finally, the choice-outcome contingencies are treated as latent variables $\rho_{s^{1},s^{2}}$ which have to be learned over the course of the experiment. We use a vague Dirichlet prior over choice-outcome contingencies. Inverting the generative model of outcomes using variational inference defines the inference and learning component of the behavioral model. In turn, marginal beliefs about latent states $s_{t}^{1},s_{t}^{2}$ and parameters $\rho_{s^{1},s^{2}}$ are used to define action selection, that is compute the choice likelihoods using the expected free energy (Equation 18).

Here, we use the semi-Markov representation of task dynamics within the behavioral models to define an agent that can learn latent temporal structure, form beliefs about moments of change, and anticipate state changes. We implemented the learning of the hidden temporal structure of reversals as a variational inference scheme, where we assume that the agent entertains a hierarchical representation of the reversal learning task, with a finite set of models of possible temporal structure of the dynamic environment. In other words, we assume that human brain entertains a set (possibly a very large set) of temporal templates. In Figure 4, we show the graphical representation of the generative model of behavior, which is described detail in Section 4.6. Here we will briefly introduce the relevant parametrization of the behavioral model, which are critical for understanding the model comparison results presented in the next subsection.

Each temporal template *m* corresponds to a pair of parameters *m* = (μ, ν) that define the frequency of reversals μ and the regularity of reversals ν (the higher the value the more regular the changes are). In **Figure 12**, we illustrate three of these templates, which differ in their regularity parameter ν, but all have the same frequency parameter μ. It is important to note that when ν = 1 (the lowest value) the temporal templates correspond to the hidden Markov model (HMM) representation. HMM representation implies that the moments of reversals are unpredictable, or maximally irregular. Here we use the HMM representation as a reference point for determining whether participants were able to learn latent temporal structure of reversals, and whether they a priori expected predictable moments of reversal.

When simulating behavior and fitting the model to participants' choices, we use a prior probability *p*(*m*) over temporal templates *m* to restrict otherwise rich set of all possible temporal templates *m* = (μ, ν), that span all combinations of μ ∈ {5, …, 45} and ν ∈ {1, …, 10}. Hence, template prior *pm* reflects prior expectations of an agent at the beginning of the experiment about the possible temporal structure of the task dynamics. Therefore, to capture a wide range of prior beliefs we require a flexible prior *p*(*m*) that can reflect subjects with different prior expectations about temporal structure. Posterior estimates of the most likely parameterizations of the temporal prior, allows us to infer from the behavioral data if participants' beliefs are a priori precise and biased toward expecting irregular reversals, or are imprecise and accommodate a wide range of possible latent temporal structures. In the model, we use the following prior over temporal templates:


(1) $$\begin{array}{l} p\left(m|\nu_{max}\right)=p\left(\mu ,\nu |\nu_{max}\right)\\ \;=p(\mu )p\left(\nu |\nu_{max}\right)\\ \;p(\mu )=\frac{1}{40}\\ p\left(\nu |\nu_{max}\right)=\{\begin{array}{cc} \frac{1}{\nu_{max}} & \text{ for }1\leq \nu \leq \nu_{max}\\ 0 & \text{ otherwise} \end{array} \end{array}$$

where ν_*max*_ ∈ {1, …, 10}. Note that the prior regularity parameter ν_*max*_ reflects Bayesian prior expectations about the maximal precision of between-reversal intervals. In other words, ν_*max*_ captures the agent's expectations about the maximal regularity of reversals, and hence their predictability. Thus, with this parameterization we assume that subjects, at the beginning of the experiment, have uniform beliefs about a possible mean duration between reversal interval, but might differ in their propensity to represent high or low regularity of between-reversal intervals. For example, some subjects could hold precise beliefs that reversals were not under their control and were therefore inherently unpredictable (corresponding to ν_*max*_ = 1). Such a subject would fail to learn—or accumulate evidence for—the regularity of reversals in the regular condition. Conversely, some participants may have imprecise prior beliefs about regularity (ν_*max*_ > 1); enabling them to learn that reversals were regular, thus predictable, in the appropriate condition.

The beliefs about temporal templates, influence the beliefs about the reversal probability on any given trial (i.e., how likely is that a reversal occurs in the next trial), and consequently modulate beliefs about the latent state of the task (i.e., which card is associated with high reward probability) and corresponding outcome probabilities. In turn, the beliefs about the latent state influence the choices. As mentioned above, choices are defined as the minimizers of the expected free energy (surprise about future outcomes), typically denoted by *G*. Given the expected free energy *G*_*a*_[***P***_*o*_, ν_*max*_, *t*] of action *a* on trial *t* we define the choice likelihood as


(2) $$\begin{array}{l} a_{t}\sim p\left(a\right)\propto e^{\gamma G_{a}[P_{o},\nu_{max},t]}. \end{array}$$

Here, the parameter γ denotes choice precision, the vector of probabilities $P_{o}=\left(p_{-},p_{+},\frac{1}{2}p_{c},\frac{1}{2}p_{c}\right)$ denotes prior preferences over possible outcomes, that is, losses (−), gains (**+**), and cues (c). In active inference (Friston et al., 2017) prior preference parameter ***P***_*o*_ defines a preference of the agent to observe rewards and collect information (engage with the exploratory option). This balance is at the core of active inference and rests upon choosing actions that minimize expected free energy (see Section 4). In turn, expected free energy can be decomposed into epistemic value (i.e., expected information gain) and extrinsic value (i.e., expected preferences or reward). The relative contribution of epistemic and extrinsic value depends upon the precision of preferences over outcomes. In other words, if subjects do not care which of the four outcomes they encounter, then they will behave in a purely exploratory fashion. Conversely, if they have precise or strong preferences, extrinsic value will dominate. In our setup, the precision of preferences rests on two differences; namely the difference between reward and loss, and the difference between collecting rewards or information. Interestingly, a prior preference for collecting information has, itself, epistemic affordance (or at least has greater epistemic value than collecting rewards). This kind of prior preference emerges during the formation of epistemic habits. In the terminology of reinforcement learning, the logarithm of prior preferences ln ***P***_*o*_ assigns a subjective value to possible outcomes, and the expectation of log-preferences defines the expected value of different actions (see Equation 18).

Importantly, we use Equation (2) in two different ways: (i) as a mapping from beliefs into actions which we used to simulate behavioral choices, and (ii) as a choice likelihood which we use for inverting the model when fitting the model to subjects' choices to derive the posterior estimates of free model parameters (γ, *p*_−_, *p*_+_, ν_*max*_), individually for each subject. Details of the model inversion procedure are described in Section 4.7.

#### 2.2.1. Simulating the behavioral effect of prior expectations over temporal templates

By simulating the model's behavior given different values of temporal regularity parameter ν_*max*_, we aimed to demonstrate that the agent can acquire a correct representation of the latent temporal structure in different experimental conditions, and that ν_*max*_ influences the dynamics of both performance and probing. Importantly, different values of ν_*max*_ should lead to sufficiently distinct behavior, if we hope to accurately associate subjects' behavior with underlying model parameterization.

**Figure 5:**
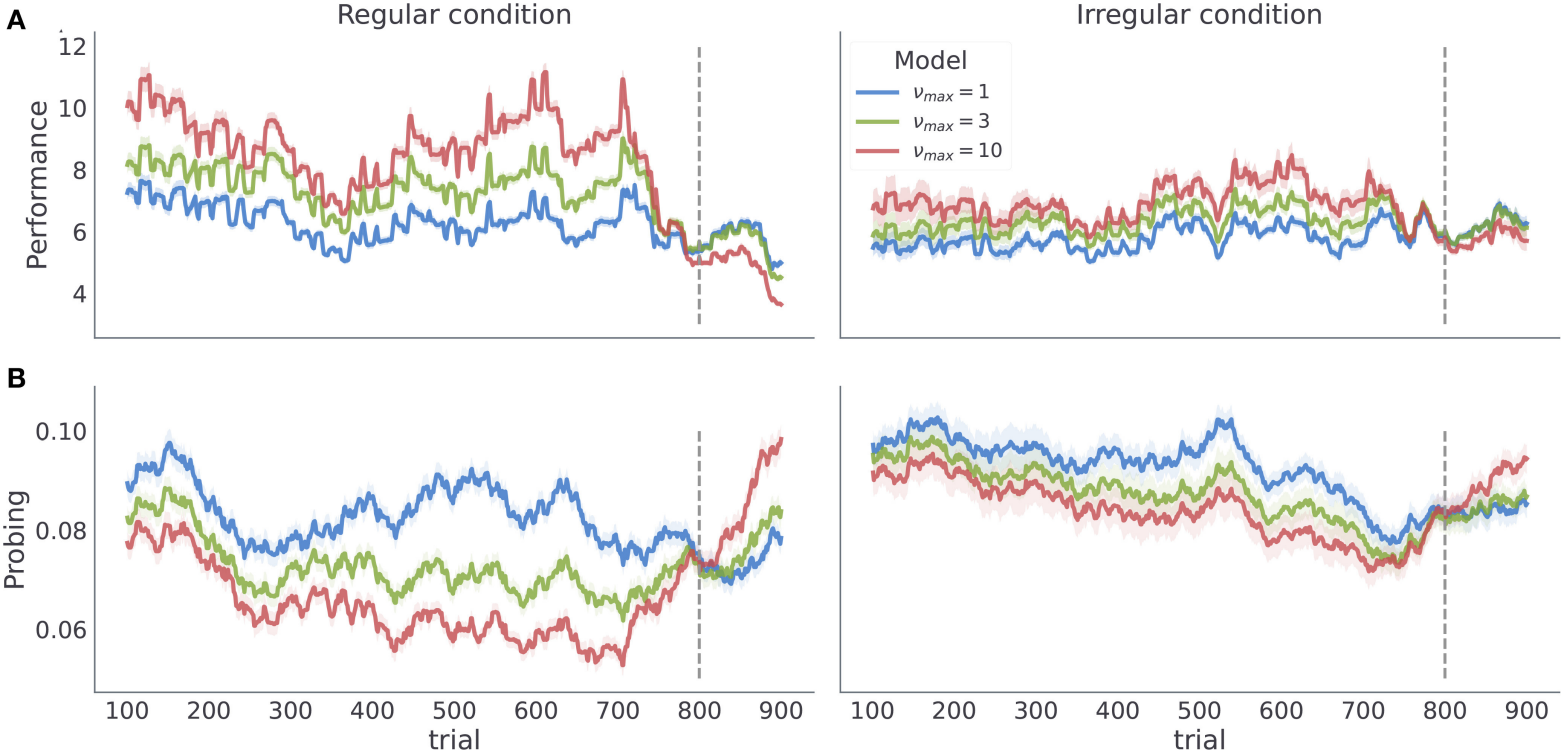
Model dependent dynamics of behavioral measures for varying ν_*max*_. Each line corresponds to an average over *n* = 50 simulated trajectories with γ = 5, and ***P***_*o*_ = (0.1, 0.6, 0.15, 0.15). **(A)** Performance estimated as odds of generating a correct choice within a 200 trials long time window centered at trial index. **(B)** Probing, computed as odds of selecting the exploratory option within the 200 trials long time window. The shaded colored areas around the trajectories correspond to the 95% confidence interval.

The temporal regularity parameter ν_*max*_ is the key parameter in the model to understand how learning about temporal structure comes about. As ν_*max*_ constrains the maximal temporal regularity the agent expects in the task, it is a measure of subjects' sensitivity to the latent temporal structure. Importantly, we find that varying ν_*max*_ results in simulated behavior with distinct behavioral patterns in our two experimental conditions as shown in Figure 5. As we increase ν_*max*_ the behavioral performance increases, in both conditions. In contrast, as we increase ν_*max*_ the probing decreases, as the agent is more certain about the moment of reversal and requires information provided by exploratory option less often. Note that different values of ν_*max*_ induce stronger differences in both performance and probing in the regular condition, compared to the irregular condition. Practically, this means that we can infer ν_*max*_ from behavioral data with higher precision in regular than in irregular condition. We validate the classification accuracy of ν_*max*_ based on posterior estimates given simulated data in the form of confusion matrix as shown in Supplementary Figure 2. Note that even in the ideal case when behavior is generated exactly from the behavioral model, classification accuracy with regard to ν_*max*_ is substantially lower in irregular compared to irregular condition. We will clarify the impact of low classification accuracy in the next subsection when discussing the results of model-based analysis.

#### 2.2.2. Demonstrating the learnability of latent temporal structure

**Figure 6:**
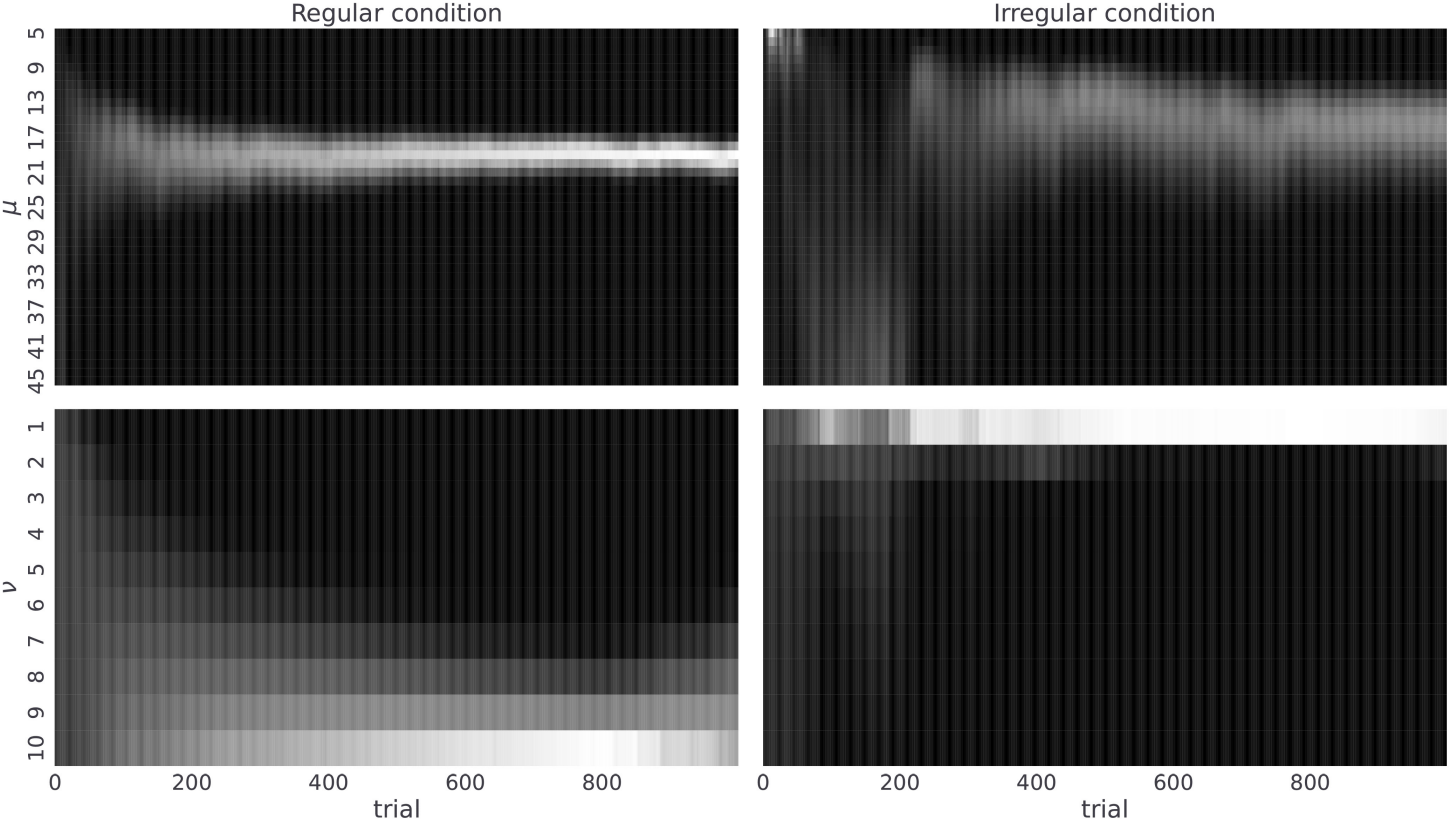
Posterior beliefs about temporal templates. Posterior beliefs of a single agent in the regular **(left)** and the irregular condition **(right)**. Posterior beliefs *q*_*t*_(*m*) = *q*_*t*_(μ, ν) at each trial *t* over templates *m* are marginalized over precision parameter ν obtaining *q*_*t*_(μ) **(top)** and mean parameter μ obtaining *q*_*t*_(ν) **(bottom)**. The posterior beliefs are estimates obtained from a single run of the agent in both experimental conditions where we fixed the temporal prior parameter to ν_*max*_ = 10, choice precision to γ = 5, and the preference vector to ***P***_*o*_ = (0.1, 0.6, 0.15, 0.15). The lighter the color the higher is the corresponding posterior probability for that parameter value.

As a next step we will illustrate that the agent with the highest value of temporal prior (ν_*max*_ = 10)—that is, the agent with the most adaptable beliefs about the latent temporal structure—is capable of accurately inferring the correct temporal template *m*, and that the rate at which agent learns correct representations of the temporal structure depends on the given temporal context. Hence, we expect that human subjects, with similar prior expectations about temporal structure, should also be capable of learning the correct statistics. In Figure 6, we show posterior beliefs over temporal templates in the form of marginal posterior beliefs about the mean μ and the regularity ν at each time step of the experiment. We see that the agent quickly learns the correct mean between-reversal duration (already after 200 trials the highest posterior probability is close to μ = 19), but it takes longer (more than 400 trials) to form precise beliefs about the level of temporal regularity. In contrast, in the irregular condition, learning the correct mean between-reversal-interval (fixed to μ = 19 in both conditions) takes more time and is less precise, but the posterior estimates over the precision parameter (ν) converge faster to the correct values (already after 200 trials). Note that having the correct representation of both mean and precision parameters is more important in the regular condition as one can achieve higher improvements in the performance compared to the irregular condition, as we demonstrated previously in Marković et al. (2019).

#### 2.2.3. Simulating the behavioral effect of prior preferences over outcomes

**Figure 7:**
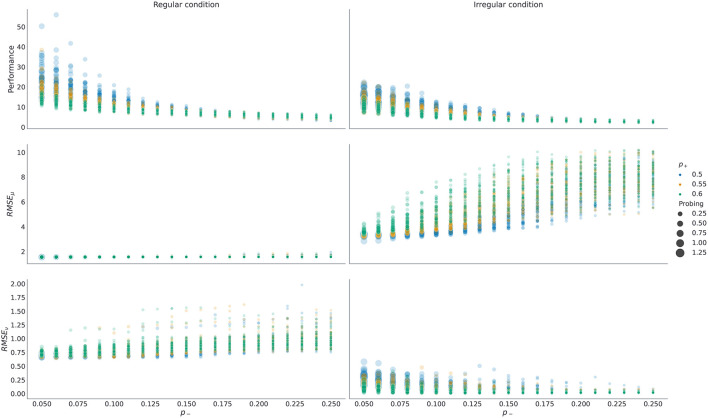
Dependence of performance, probing, and the quality of temporal representations on prior preferences over outcomes. Each dot in the plot corresponds to a single run with fixed prior preferences $P_{o}=\left(p_{-},p_{+},\frac{1}{2}p_{c},\frac{1}{2}p_{c}\right)$, with temporal prior set to ν_*max*_ = 10, and with decision precision set to γ = 5. For each possible pair ((*p*_−_, *p*_+_) ∈ {0.05, 0.06, …, 0.25} ⊗ {0.5, 0.55, 0.6}) we have repeated *n* = 50 simulations in each condition. Here *RMSE*_μ, ν_ stands for the root mean square error of corresponding parameters μ, ν that define temporal template. The RMSE is computed using posterior probabilities *q*_*t*_(μ, ν) obtained at trial *t* = 800. The performance and the probing are computed as averages over responses from trial *t* = 400 until trial *t* = 800. Note that probing is increasing as we reduce *p*_−_ and keep *p*_+_ fixed (circles of the same color), and as we reduce *p*_+_ and keep *p*_−_ fixed, as the larger the sum (*p*_+_ + *p*_−_) is, the lower is the preference for choice cues *p*_*c*_, and hence the tendency of the agent to engage with the epistemic option. Higher probing (larger circle size) results in higher performance in both conditions (top row). Similarly, both *RMSE*_μ_ and *RMSE*_ν_ are lower for larger exploration odds, with the exception of *RMSE*_ν_ in irregular condition. Note that forming an accurate temporal representation is especially important in the regular condition, where forming correct anticipatory beliefs can substantially improve behavioral adaptation and simplify the problem of balancing between exploratory and exploitative choices. In contrast, in the irregular condition, having a precise representation of temporal structure does not impact performance substantially, and the agent performs better when engaging with the epistemic option more often.

As mentioned above, the prior preference over outcomes ***P***_*o*_ parameterize agents' motivation to collect rewards (generate correct choices) and collect information (engage with the exploratory option). Therefore, it is important to understand how prior preferences interact with performance and probing. We show that the more an agent engages with the exploratory options (i.e., the higher its preference for choice cues), the better its representation of latent temporal structure, and consequently the higher agent's performance. This is because selecting exploratory options maximally reduces the uncertainty about the latent state (which option has higher reward probability) which in turn allows an agent to learn a more accurate representation of the latent task dynamics. We visualize these dependencies in Figure 7, where we show what impact changing *p*_+_ and *p*_−_ have on performance, probing, and the quality of temporal representation after 800 trials. In the Supplementary Figure 3 we show the same dependencies but with respect to changing *p*_+_ and *p*_*c*_, hopefully helping the reader to build an intuition about interactions between prior preference parameter and behavior. Note that in both figures we only consider cases in which *p*_+_ ≥ *p*_−_ as this reflects higher prior preference for gains than for losses in the agent, which we expect to hold for all subjects.

### 2.3. Model-based analysis of subjects' choices

By estimating the prior beliefs—under a semi-Markovian generative model—that best explain observed choice behavior, we next ask whether human subjects can learn latent temporal regularities in the reversal learning tasks? An individual's capacity to learn correct temporal regularity corresponds to their behavior being associated with a less precise prior over temporal templates (Equation 1), that is, larger ν_*max*_. An agent with imprecise prior over temporal templates is able to learn an accurate representation of a distribution of between-reversal-intervals, and to form expectations about the moment of reversals (see Figures 6, 7) in both conditions. Thus, we anticipated that between-subject variability in performance and probing would be reflected in different posterior estimates of the most likely ν_*max*_ value associated with the behavior of individual subjects.

**Figure 8:**
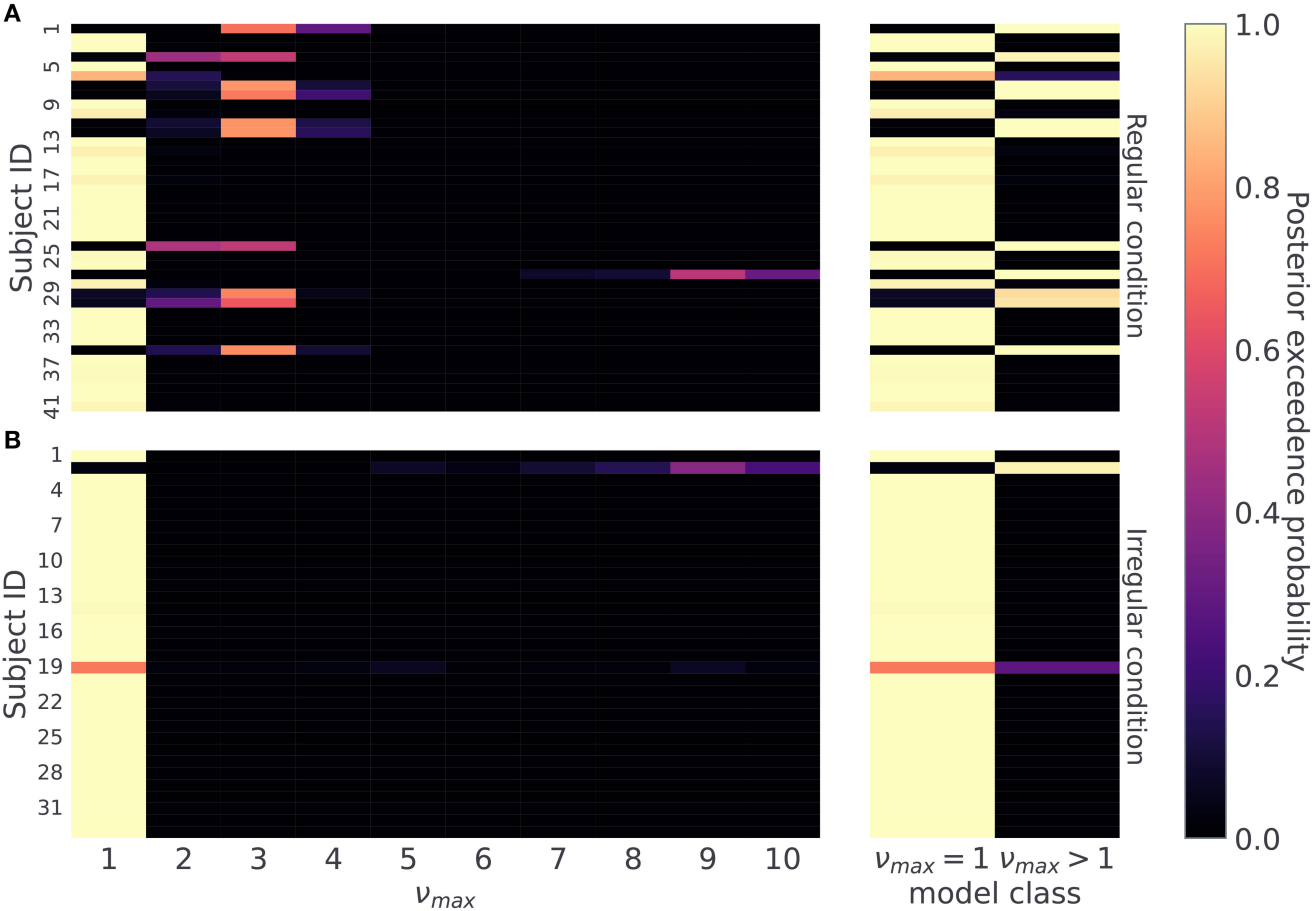
Posterior probability over temporal prior ν_*max*_. Posterior probability of possible ν_*max*_ values for each subject, reflecting a subject's flexibility to learn latent temporal structure: **(A)** regular condition, and **(B)** irregular condition. On the right hand side, we combine posterior estimates into two classes, one for the limiting case ν_*max*_ = 1, and another for all other options ν_*max*_ > 1. This split differentiates subjects not sensitive to temporal regularities from the ones who a priori expected a regular temporal structure of reversals. Note that lighter colors correspond to higher posterior probability.

Therefore, we first classify subjects based on the maximum a-posteriori estimate over possible values of ν_*max*_ ∈ {1, …, 10}, as shown in Figure 8. For each subject we compute a posterior probability over ν_*max*_ and assign the subject the value of the temporal prior ν_*max*_ corresponding to the value with the highest exceedance probability (see Section 4.7). Using this procedure we find that 11 out of 41 subjects in the regular condition, and 1 out of 33 subjects in the irregular condition are assigned to the group with temporal prior ν_*max*_ > 1. For the subjects in the regular condition this result suggests that about a quarter of subjects learned to anticipate reversals to a certain extent. As our aim is not to identify precisely participants' temporal prior, but simply to distinguish between subjects that learn temporal regularities (ν_*max*_ > 1) from those that do not (ν_*max*_ = 1), limiting the analysis to binary classification leads to the following classification accuracy in simulated data: (i) in the regular condition ν_*max*_ = 1, *ACC* = 1, and ν_*max*_ > 1, *ACC* = 1, (ii) in the irregular condition ν_*max*_ = 1, *ACC* = 1.0 and ν_*max*_ > 1, *ACC* = 0.9. Note that in regular condition we have around 10% chance of misclassifying a subject that actually has a less precise prior over temporal templates (ν_*max*_ > 1).

**Figure 9:**
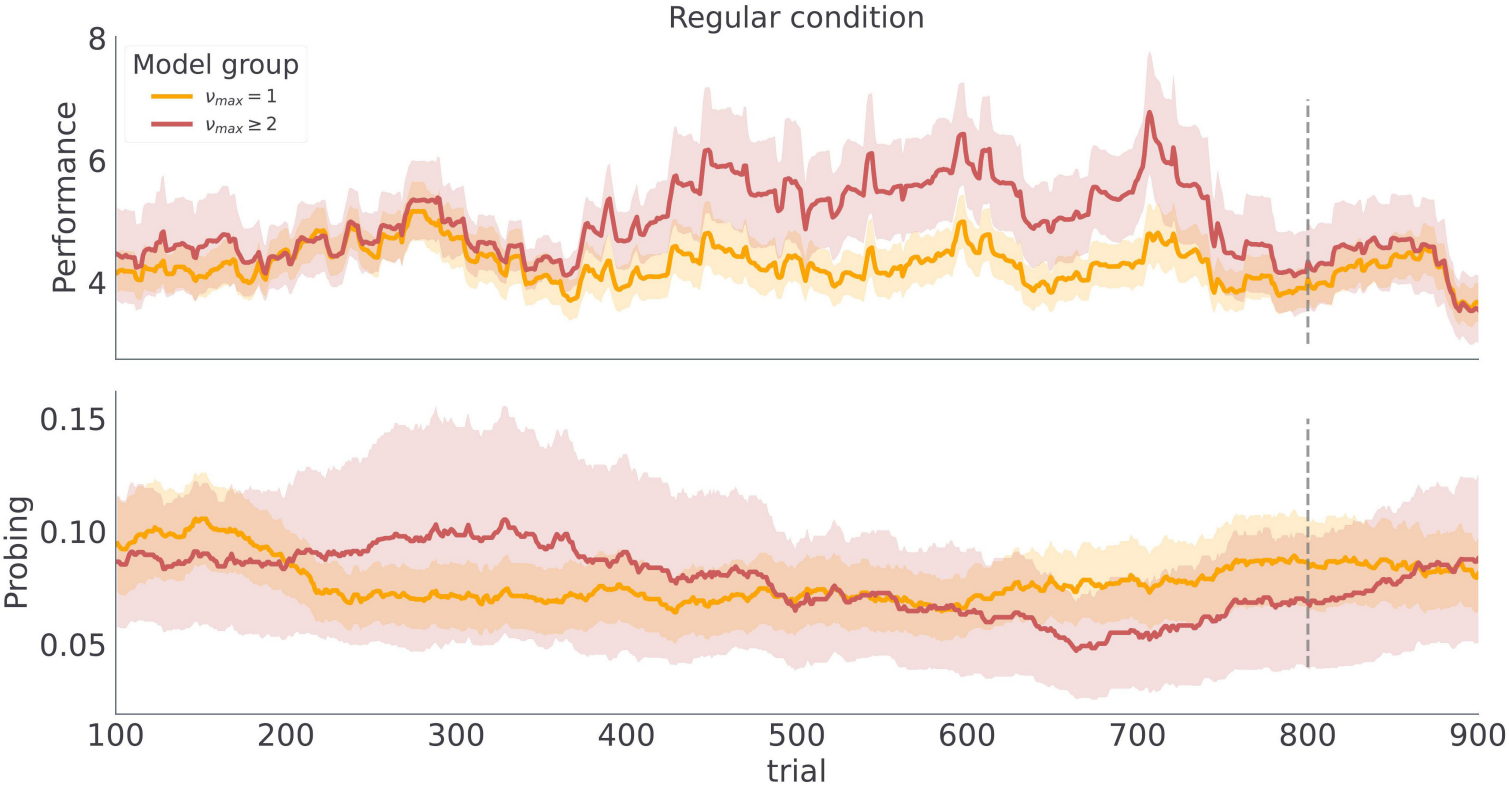
Category based mean estimate of behavioral measures. Each line corresponds to a model class average over behavioral trajectories of subjects assigned to that model class. Note the similarity of the trajectory profiles to the simulated trajectories in Figure 5 regular condition. The shaded colored areas around the trajectories correspond to the 95% confidence interval.

The posterior estimates of model parameters shown in Figure 8 show that the majority of participants were assigned to the model class corresponding to the simplest HMM representation (ν_*max*_ = 1) which assumes maximal irregularity. However, in the regular condition we also find a number of participants (27%) that exhibit more flexible priors, allowing us to form two subject groups. Importantly, when we plot the time course of both performance and probing, as shown in Figure 9, we find a trajectory of behavioral measures over the course of the experiment similar to what we see in simulated data. Namely, that the performance is higher and the probing reaches lower values in the group of participants associated with larger ν_*max*_ (compare with Figure 5—regular condition). We excluded the irregular condition from the visualization as we did not find sufficient number of subjects with associated with *nu*_*max*_ > 1. The behavioral trajectories of individual participants are shown in Supplementary Figure 1.

**Figure 10:**
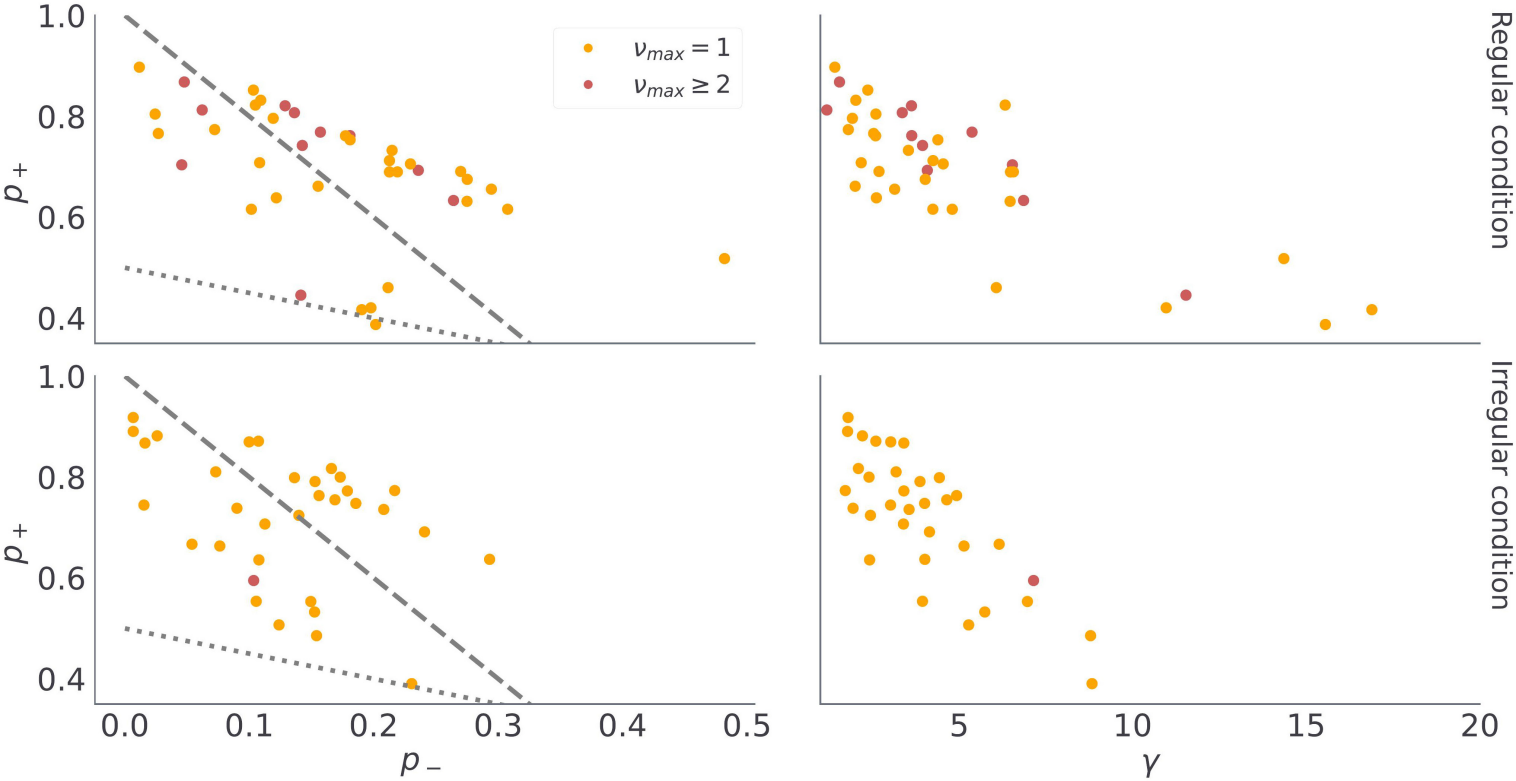
Posterior median of continuous model parameters. Each dot corresponds to the median of the *n* = 1, 000 samples from the posterior distribution of γ, *p*_−_, and *p*_+_ for each participant. The dashed gray lines denotes equality between preferences for losses and epistemic cues, that is, when $p_{-}=\frac{p_{c}}{2}$ then *p*_−_ = (1 − *p*_+_)/2. In turn, the doted grey line indicates equality between preferences for gains and epistemic cues, that is, when $p_{+}=\frac{p_{c}}{2}$ then *p*_−_ = 1 − 3*p*_+_. Note that we use the same color coding as in the previous figure to denote classification of participants into different model classes.

These findings show a good correspondence between simulated behavior for different parameterizations of the model (ν_*max*_ = 1 vs ν_*max*_ > 1 in Figure 5), and the participants' behavior associated with different model classes (Figure 9). There are two possible explanations for this: (i) the model inversion accurately captures the participants behavior and between-participant sensitivity to temporal regularities of the task, (ii) the group differences come from other free model parameters and do not correspond to differences in sensitivity to temporal structure. To exclude the second option we show in Figure 10 the mean of the posterior estimates of free model parameters γ, *p*_−_ and *p*_+_. Note that in both experimental conditions we see a lack of separation between free model parameters associated with each model class.

There are a couple of interesting observations to be made from the posterior expectations of the free model parameters. First, we find in most participants rather large posterior estimates of choice precision γ, close to γ = 5 (see Figures 10B,D), suggesting that choice stochasticity is rather low in most participants. Low choice stochasticity means that choices are well aligned with the choice likelihoods encoded in terms of expected free energy (Equation 18). In other words, the chosen option is the option that minimizes expected free energy and the model is rather accurate in predicting behavioral responses. Second, the posterior estimates of outcome preference parameters *p*_−_, and *p*_+_ split subjects in two distinct groups, which correspond to their preference for receiving informative cues when selecting exploratory option. The 29 subjects who never engaged with the exploratory option have a higher preference for losses than for informative cues, hence *p*_−_ ≥ *p*_*c*_. We marked with the dashed gray line (Figures 10A,C) the limiting case of $p_{-}=p_{c}=\frac{1-p_{+}}{2}$, which separates the subjects which did not interact with the exploratory option (above the dashed line) and subjects that were relying on exploratory option to reduce their belief uncertainty (below the dashed line). Similarly, participants who prefer informative cues over gains would have prior preferences over cues in the region *p*_*c*_ ≥ *p*_+_. The dotted gray line (Figures 10A,C) marks the limiting case of $p_{+}=p_{c}=\frac{1-p_{-}}{2}$. Note that only one subject in the irregular condition, and several subjects in the regular condition fall along this line.

## 3. Discussion

Sequential activity of neuronal assemblies is one of principled neuronal operations that support higher level cognitive functions (Eichenbaum, 2014; Buzsáki and Llinás, 2017) and allow humans to form complex spatio-temporal representation of our every day environment (Frölich et al., 2021). Akin to grid cells known to support representation of both spatial and non-spatial task states (Fu et al., 2021), time cells have been linked to temporal representation of state sequences critical for memory and decision-making (Eichenbaum, 2014). Importantly, in spite of these fruitful experimental findings we have no clear computational understanding of how humans learn temporal structure in the service of successfully behavioral adaptation.

Here we introduced a novel computational model of behavior capable of learning latent temporal structure of a probabilistic reversal learning task with multiple reversals (Costa et al., 2015; Reiter et al., 2016, 2017; Vilà-Balló et al., 2017). The computational model combines hidden semi-Markov framework for representing latent temporal structure (Yu, 2015) and active inference for resolving exploration-exploitation trade-off (Friston et al., 2015, 2016). Crucially, the model can be used for investigating decision making in changing environments in any behavioral task that can be cast as a dynamic multi-armed bandit problem (Gupta et al., 2011; Markovic et al., 2021); of which the reversal learning tasks is a special case corresponding to a specific type of two-armed bandit problem.

The probabilistic reversal learning task, which we utilized to demonstrate flexibility of proposed model, is one of the most established paradigms for investigating human behavior in changing environments and quantifying cognitive disorders. We used model-based analysis of behavioral data to infer temporal expectations of subjects exposed to one of the two task variants: (i) with regular intervals between reversals, (ii) with irregular intervals between reversals. Notably, being able to form expectations about the moment of reversal is critical for achieving high performance in the probabilistic reversal learning task, which we illustrate using simulations. We demonstrated that participants behavior is highly heterogeneous reflecting the differences in participants expectations about temporal regularities. Crucially, the participants expectations about temporal regularities influence their ability to correctly learn latent temporal structure (especially relevant in the condition with regular between reversal intervals), and is reflected in their performance throughout the experiment.

We have extended the standard reversal learning task and incorporated an explicit exploratory option in addition to the two standard options whose choice results in monetary gain or loss. This exploratory option informs the participant about the currently correct choice. The additional behavioral response provides us with more direct access to the individual uncertainty about a correct choice and improves model selection. Interestingly, in addition to participants' diversity of temporal representation, we find stark differences in their preferences to engage with the exploratory option, suggesting individual differences for the value of information (Niv and Chan, 2011) and utilized strategies for resolving the exploration-exploitation trade-off. Critically, their epistemic preferences are not obviously correlated with the quality of the learned temporal structure, as in both groups participants show heterogeneous prior expectations about temporal regularities limiting the available temporal templates, hence the accuracy of temporal representations. However, the willingness to engage with the epistemic options does influence participants' performance, where higher engagement results in better performance. Therefore, these joint findings reveal distinct components of the computational mechanisms that underlie adaptive behavior in dynamic environments.

To recapitulate, we have effectively shown that it is possible to explain a subject's choice behavior in terms of their prior beliefs about temporal regularity, that is, a set of temporal templates they entertain, and other contingencies that characterize the (generic) paradigm at hand. This is potentially important because this kind of phenotyping could be deployed in a neurodevelopmental or psychiatric context to summarize any subject in terms of a small number of interpretable priors. Theoretically, this sort of phenotyping provides a sufficient description of a subject *via* the complete class theorem. The complete class theorem says that for any given pair of reward functions and behaviors there exists some priors that render the behavior Bayes optimal (Wald, 1947; Brown, 1981). To be Bayes optimal is to conform to the belief updates and action selection described by active inference. This means that there is always a set of prior beliefs that provide a sufficient account of any subject specific behavior.

Having said this, we have only explored a small subset of possible sets of temporal templates. We could apply the same technology (i.e., model inversion) to ask more general questions. For example, if some subjects a priori exclude from the templates the possibility of irregular reversals. There are other priors we could have explored that place various constraints on belief updating or divergences from particular prior beliefs. These might be interestingly related to notions of motivation, cognitive effort and resources in cognitive science (Pezzulo et al., 2018); however, this would require a specification of motivation, resources and effort in terms of belief updating, which is an outstanding challenge. Overall, we expected that, as we humans are exposed to predictable changes in our everyday environment, that there should be profound evidence that subjects utilize a higher order (semi-Markovian) model. The fact that we do not see that in the model selection results (in the regular condition the majority of subjects' behavior can be associated with the simplest Markovian assumption) suggests that a better experimental paradigm than the currently used reversal learning task is required. This paradigm should be more engaging and intuitively linked to distinct latent temporal regularities. A notable limitation of the current experimental paradigm is that it is not obvious to subjects that anticipating reversals can improve performance, or that potential performance improvement is sufficiently large to justify added effort required to keep track of higher-order statistics.

To accurately predict future it is critical not only to know that change might be coming but also when the change will occur. To anticipate the changes in our-everyday environments and adapt our behavior accordingly, it is critical to accurately estimate and represent elapsed time between relevant events. Although the presented model abstracts elapsed time as a hierarchically structured counting process, it is straightforward to model events duration in physical time, by using continues representation of the phase-type distribution. This way the underlying model corresponds to continues time semi-Markov processes (Hongler and Salama, 1996) where state transitions follow the master equations, allowing one to capture decision making in real-time. Notably, an implicit assumption we make here is that a simple counting process can represent elapsed time at multiple time scales. In fact, various experimental findings suggest that the brain employs counting mechanisms, represented over multiple timescales, and integrates those representations when generating behavior (Baldassano et al., 2017; Fountas et al., 2022). Similarly, a range of experimental findings has linked timing of events and hence forecasting the future to underlying Bayesian inference mechanisms (Jazayeri and Shadlen, 2010; Griffiths and Tenenbaum, 2011). Most recently, Maheu et al. (2022) has linked sequence learning and prediction in human subjects to an underlying hierarchical Bayesian inference model with distinct hypothesis spaces for statistics and rules corresponding to a set of deterministic temporal templates. The authors conclude that the hierarchical Bayesian inference mechanism underlies human ability to process sequence, similar to hierarchical semi-Markov framework proposed here.

Furthermore, in recent years, various neuroimaging studies have linked different neuro-cognitive domains, such as attention and working memory, to specific spatio-temporal expectations about underlying dynamics of the environment (Nobre and Van Ede, 2018). Interestingly, the human ability to estimate and reproduce elapsed time was also previously linked to reward discounting and intertemporal choice behavior (Ray and Bossaerts, 2011; Retz Lucci, 2013; Bermudez and Schultz, 2014). For example, McGuire and Kable (2015) demonstrated that “impulsivity” (reluctance to wait for a better reward), depends on the hidden statistics of delays—between an initial bad offer and a later but more valuable offer—which human participants experienced. Using a similar “limited offer” game (with a constant latent temporal statistics) and active inference representation of behavior (Schwartenbeck et al., 2015) have linked the dopaminergic midbrain activity with expected certainty about desired outcomes. In Mikhael and Gershman (2019), the authors have linked time perception and dopaminergic neuronal activity, demonstrating the role of value-based prediction errors in time representation. Furthermore, time perception and timed behavior have been linked to all major neuromodulatory systems (Meck, 1996) either directly using neuropharmacological manipulations (Crockett and Fehr, 2014) or indirectly using neurological disorders (Story et al., 2016) and aging research (Read and Read, 2004).

Together these findings provide important evidence for the role of temporal expectations in goal-directed decision making and let one speculate whether a range of aberrant behaviors might be related to an erroneous representation of the temporal structure of the task. Importantly, the computational behavioral model that we introduced here can emulate the learning of temporal structure, hence can become a potent tool linking aberrant behavior found in cognitive disorders to erroneous prior beliefs about the rules that govern the dynamics of the environment, as suggested by the active inference account of human behavior (Friston et al., 2015, 2016, 2017).

To conclude, the results presented here provide novel insights into computational mechanism underlying the human ability to learn hidden temporal structure of the environment and the computational principles they utilize for making decisions based on temporal representations. The fact that we find behavioral heterogeneity in a population of healthy young adults suggests a potential use of the proposed design and behavioral model for cognitive phenotyping and for revealing causes of aberrant behavior in clinical populations.

## 4. Methods and materials

### 4.1. Code availability statement

All code for reproducing the figures and running data analysis and simulation algorithms is available at https://github.com/dimarkov/pybefit.

### 4.2. Experiment

#### 4.2.1. Probabilistic reversal learning

In the experimental task subjects were deciding between two cards shown on a screen, each showing a different stimulus (a geometric shape, e.g., rectangle, triangle, or a question mark) as shown in Figure 1. The reward probabilities associated with the two choice options were anti-correlated on all trials: whenever reward probability of choice A was high (*p*_*H*_ = 0.8) the reward probability of choice B was low (*p*_*L*_ = 0.2), and vice versa. Note that *p*_*H*_ = 1 − *p*_*L*_ on all trials. The location of each stimulus on the screen (right or left side) was kept fixed over trials. After each choice the stimulus was highlighted and depicted for 1.5s minus the reaction time. The feedback in the form of a gain or a loss was shown for 0.5s. Similarly, the feedback after an exploratory choice was also shown for 0.5s. If no response occurred during the decision window of 3s, the message “too slow” was presented, and no outcome was delivered.

All subjects underwent a training session during which they had the opportunity to learn the statistics of the rewards associated with high *p*_*H*_ and low *p*_*L*_ reward probability choices. The set of stimuli used in the training phase differed from the one used during the testing phase. Subjects were instructed that they could either win or lose 10 cents on each trial, and that they will be paid the total amount of money they gained during the testing phase at the end of the experiment. Each subject performed 40 training trials with a single reversal after the 20th trial. Before the start of the testing phase subjects were told that the reward probabilities might change at regular intervals (in both conditions) over the course of the experiment. No other information about reversals or the correlation of choices and outcomes was provided. Thus, the subjects had no explicitly instructed knowledge about the anti-correlated reward probabilities or between-reversal-intervals before the experiment.

Note that, out of *n* = 74 participants *n*_*p*_ = 24 were exposed to the variant of the reversal learning task without epistemic option. This group of subjects belongs to an initial pilot study that used the standard two-choice task design. In the pilot study 14 subjects were assigned to the regular condition and 10 to the irregular condition. We decided to include the subjects from the pilot into the analysis, as we noticed that almost 30% of subjects, in the post pilot group, choose not to interact at all with the exploratory option, even when that was a possibility. We will not explore this finding here in more detail, but we can exclude their misunderstanding of the task as a potential confound, as we provided a detailed instructions and training before they performed the task (see Section 4.3 for more details).

### 4.3. Behavioral measures

To quantify behavior we have used two summary measures: (i) performance, defined as odds of making a correct choice, and (ii) probing, defined as odds of making an exploratory choice.

**Figure 11:**
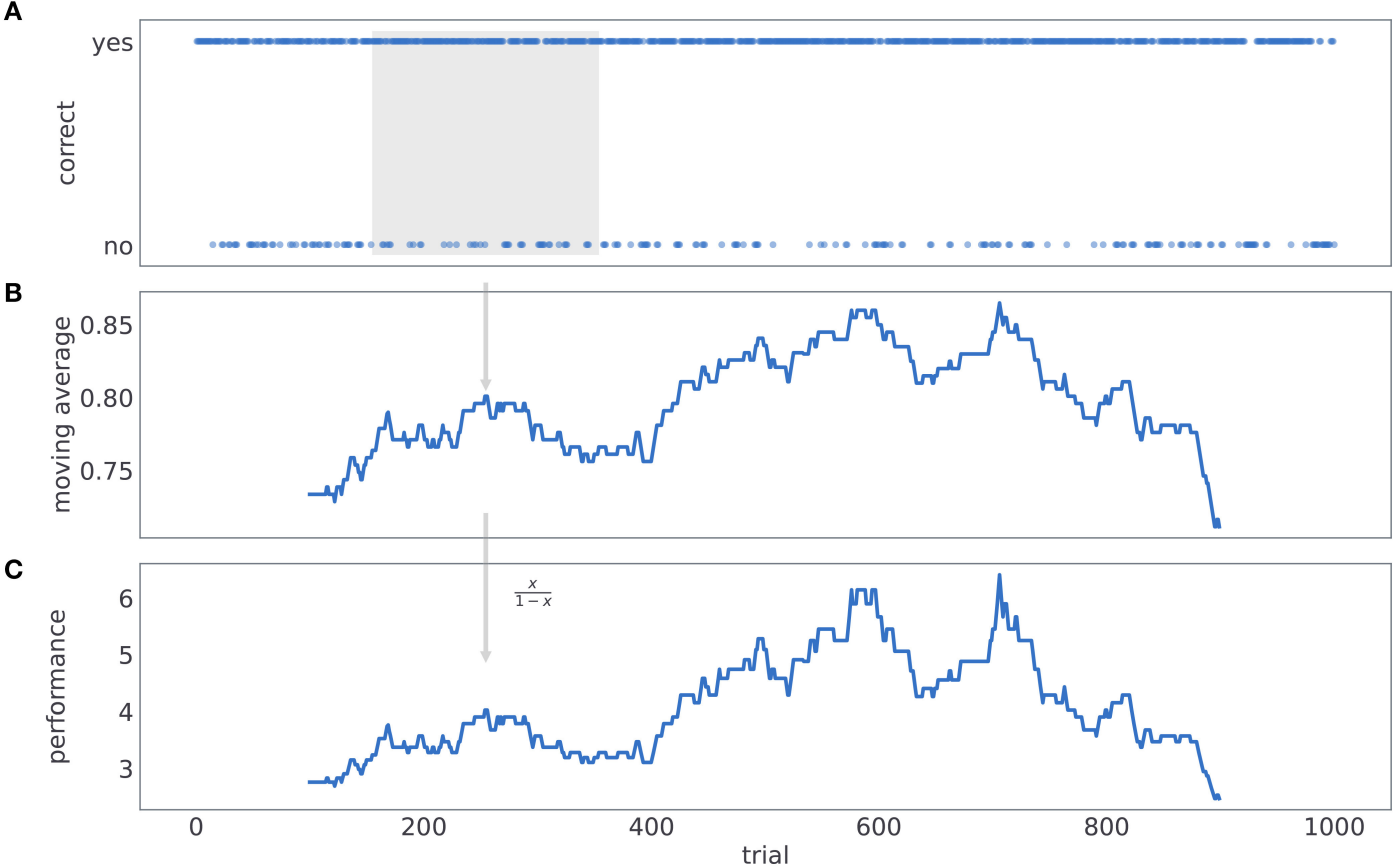
Computing behavioral performance. The process of computing *performance*. **(A)** We label subjects' responses as either correct or incorrect, depending on whether a card with higher reward probability was selected or not. **(B)** We compute a probability of making a correct choice within a 200 trial window, centered at the current trial number *t*. **(C)** For each trial we compute performance as odds of being correct.

The process of computing *performance* is illustrated in Figure 11. We first label subjects' responses as either correct or incorrect, depending on whether a card with higher reward probability was selected or not (see Figure 11A). Then we compute a probability of making a correct choice within a 201 trial window, centered at the current trial number *t* (see Figure 11B). Finally, for each trial we compute performance as odds of being correct (see Figure 11C).

The *probing* is computed in similar manner to performance, with the only difference that we label choices as either exploratory or exploitative depending on whether subjects have chosen the exploratory option (middle card in Figure 1), or not. Probing is defined as the odds of selecting the exploratory option within a 200 trials time window.

### 4.4. Behavioral model

To introduce the generative model of task dynamics, and subsequently derive the behavioral model *via* model inversion methods, we will consider the following features of the task. At any trial the task environment is in one of the two possible states, defined as the configuration of reward contingencies. For example, state one corresponds to stimulus A being associated with a high reward probability *p*_*H*_, and state two to stimulus B being associated with a low reward probability *p*_*L*_. Subjects do not know in advance how likely rewards and losses are when making a correct choice compared to making an incorrect choice, and this is something they have to learn during the course of experiment. In other words, we also treat reward probabilities (*p*_*H*_ and *p*_*L*_) as latent variables. Between trials the state can change, i.e., when a reversal occurs but only after a certain minimum number of trials has elapsed since the last state change. Depending on the experimental condition the between reversal duration will either be semi-regular (occurring every 20 trials with small variability) or irregular (occurring every 20 trials, but with maximal variability)

The explicit representation of state duration *d* enables us to associate changes in state transition probabilities with the current trial and the moment of the last change. The dependence of state transition probability on the number of trials since the last change corresponds to the formalism of hidden semi-Markov models (HSMM; Murphy, 2002; Yu, 2010), which allows mapping complex dynamics of non-stationary time series to a hierarchical, time aware, hidden Markov model. However, using an explicit representation of context duration is inefficient, as it requires an enormous state space representation. Here, we will instead adopt a phase-type representation of duration distribution (Varmazyar et al., 2019) which substitutes duration variable *d* ∈ 1, …, ∞ with a phase variable *f* ∈ 1, …, *f*_*max*_, allowing for a finite state representation of an infinite duration state space.

In what follows we will define the components of the generative model (observation likelihood, the dynamics of latent variables, and the parameterization of the dynamics) and derive the corresponding update rules for latent variables and state, hence enabling the learning of different temporal contexts during the experiment. The graphical representation of the generative model is shown in Figure 4.

Practically we introduce four latent states, to describe the task on any trial:

- First, the configuration of reward contingencies can be in one of the two possible states. Hence, $s_{t}^{1}\in \{1,2\}$ which describes which card is associated with high reward probability and which with low reward probability.
- Second, choosing one of the options on a given trial corresponds to setting the task in one of the three possible choice states $s_{t}^{2}\in \{1,2,3\}$ (chosen left card, chosen middle card—exploratory option, and chosen right card) corresponding to the chosen option. The choice of the option is deterministic and this state is always known with certainty after the choice is made.
- Third, current phase *f*_*t*_ ∈ {1, …, ν + 1} of the task dynamics. The phase latent variable controls transitions of latent state $s_{t}^{1}$, where the change of state is only possible if the end phase (*f*_*t*_ = ν + 1) is active on the current trial. Note that the larger the number of phases is (parameter ν ∈ 1, …) the more regular is the occurrence of reversals. We have limited here the number of phases by setting ν = 10, as this is sufficiently large for accurate representation of reversal dynamics in regular condition.
- Fourth, temporal template *m*. Latent temporal template defines the frequency of reversals, μ (mean between-reversal duration) and the number of latent phases ν, that is the regularity of reversals.

#### 4.4.1. Observation likelihood

The observation likelihood links latent states ($s_{t}^{1}$, and $s_{t}^{2}$) with probabilities of observing different possible outcomes in those states.

In the temporal reversal learning task there are four possible outcomes: (1) loss of 10 Eurocents, (2) gain of 10 Eurocents, (3) the correct card is left card, or (4) the correct card is the right card. Therefore, we define the observation likelihood as a categorical distribution


(3) $$\begin{array}{l} p\left(o_{t}|\rho ,s_{t}^{1},s_{t}^{2}\right)=\overset{4}{\underset{i=1}{\prod }}\rho_{s_{t}^{1},s_{t}^{2},i}^{\delta_{o_{t},i}} \end{array}$$

where *i* denotes the outcome type, *o*_*t*_ ∈ {1, …, 4}. The probabilities of different outcomes are parameterized *via* $\rho_{s_{t}^{1},s_{t}^{2},i}$, where each state tuple ($s_{t}^{1},s_{t}^{2}$) corresponds to a unique probability of observing any of four possible outcomes. We define prior beliefs about outcome probabilities in the form of a product of Dirichlet distributions


(4) $$\begin{array}{l} p\left(\rho \right)=\overset{4}{\underset{s^{1}=1}{\prod }}\overset{3}{\underset{s^{2}=1}{\prod }}Dir\left(\rho_{s^{1},s^{2}}|\alpha_{s^{1},s^{2}}^{0}\right). \end{array}$$

We set the parameters of Dirichlet priors to the following values:

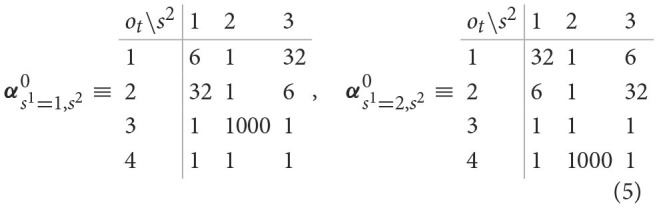



The above configuration for the parameterization of prior Dirichlet probabilities reflects an assumption that the participants have formed during training an initial—vague beliefs—about reward probabilities associated with different actions in different states. We assume that participants are highly certain that selecting the epistemic option does not return gain or loss (high value of $\alpha_{s^{1},s^{2}=2}$ for the corresponding outcome in both states). Furthermore, we assume that participants have formed good expectations gain/loss probabilities ($\langle p_{H}\rangle =\frac{32}{40}=0.8$, and $\langle p_{L}\rangle =\frac{6}{40}=0.15$), but that they are still uncertain about the exact values. Weak priors about outcome probabilities allow for ongoing adaptation of beliefs during the course of experiment.

#### 4.4.2. Hidden state dynamics

To formalize the presence of sequential reversals, we define the phase dependent state transition probability as follows


(6) $$p\left(s_{t}^{1}|s_{t-1}^{1},f_{t-1}\right)=\{\begin{array}{ll} I_{2}, & \text{if }f_{t-1}=\nu +1,\\ J_{2}-I_{2}, & \text{if }f_{t-1}\leq \nu , \end{array}$$

where *I*_2_ denotes the 2 × 2 identity matrix and *J*_2_ denotes the 2 × 2 all-ones matrix. The above relations describe a simple deterministic process for which the current state $s_{t}^{1}$ remains unchanged as long as the phase variable *f*_*t*−1_ remains below the end phase, ν + 1. The transition between states occurs with certainty (e.g., if $s_{t-1}^{1}=1$ then $s_{t}^{1}=2$) once the end phase is reached, that is, when *f*_*t*−1_ = ν + 1.

Although it is possible to condition state changes on a duration variable *d*, as demonstrated in Marković et al. (2019), such an explicit representation is inefficient as it requires large state spaces (Vaseghi, 1995; Yu and Kobayashi, 2003). Here we adopt the discrete phase-type (DPH) representation of duration distribution (Varmazyar et al., 2019). The DPH representation defines transitions between phase variables *f*_*t*_ and the following parameterization of phase transition probabilities corresponds to the DPH representation of the negative binomial distribution


(7) $$p(f_{t}|f_{t-1},m)=\{\begin{array}{ll} \delta_{m}, & \text{if }f_{t-1}\leq \nu ,\text{and }f_{t}=f_{t-1}+1\\ 1-\delta_{m}, & \text{if }f_{t-1}\leq \nu ,\text{and }f_{t}=f_{t-1}\\ \pi_{f_{t}}^{m}, & \text{if }f_{t-1}=\nu +1,\\ 0, & \text{otherwise} \end{array}$$

where $\pi_{i}^{m}=\left(\begin{array}{c} \nu \\ i-1 \end{array}\right)\;{\left(1-\delta_{m}\right)}^{\nu -i-1}\delta_{m}^{i-1}$ for *i* < ν + 1, and $\pi_{\nu +1}^{m}=1-\overset{\nu }{\underset{i=1}{\sum }}\pi_{i}^{m}$.

The corresponding negative binomial distribution of between-reversal duration can be expressed as follows


(8) $$p_{m}(d)=\left(\begin{array}{c} d+\nu -2\\ d-1 \end{array}\right)\;{\left(1-\delta_{m}\right)}^{d-1}\delta_{m}^{\nu };\;d\in \left\{1,2,\ldots \right\}$$

where the expected duration corresponds to


(9) $$\begin{array}{l} E_{p_{m}}\left[d\right]=\frac{\nu \left(1-\delta_{m}\right)}{\delta_{m}}+1=\mu +1;\;\delta_{m}=\frac{\nu }{\mu +\nu }, \end{array}$$

and variance, hence uncertainty about duration regularity, to


(10) $$\begin{array}{l} Var_{p_{m}}\left[d\right]=\mu +\frac{\mu^{2}}{\nu }. \end{array}$$

**Figure 12:**
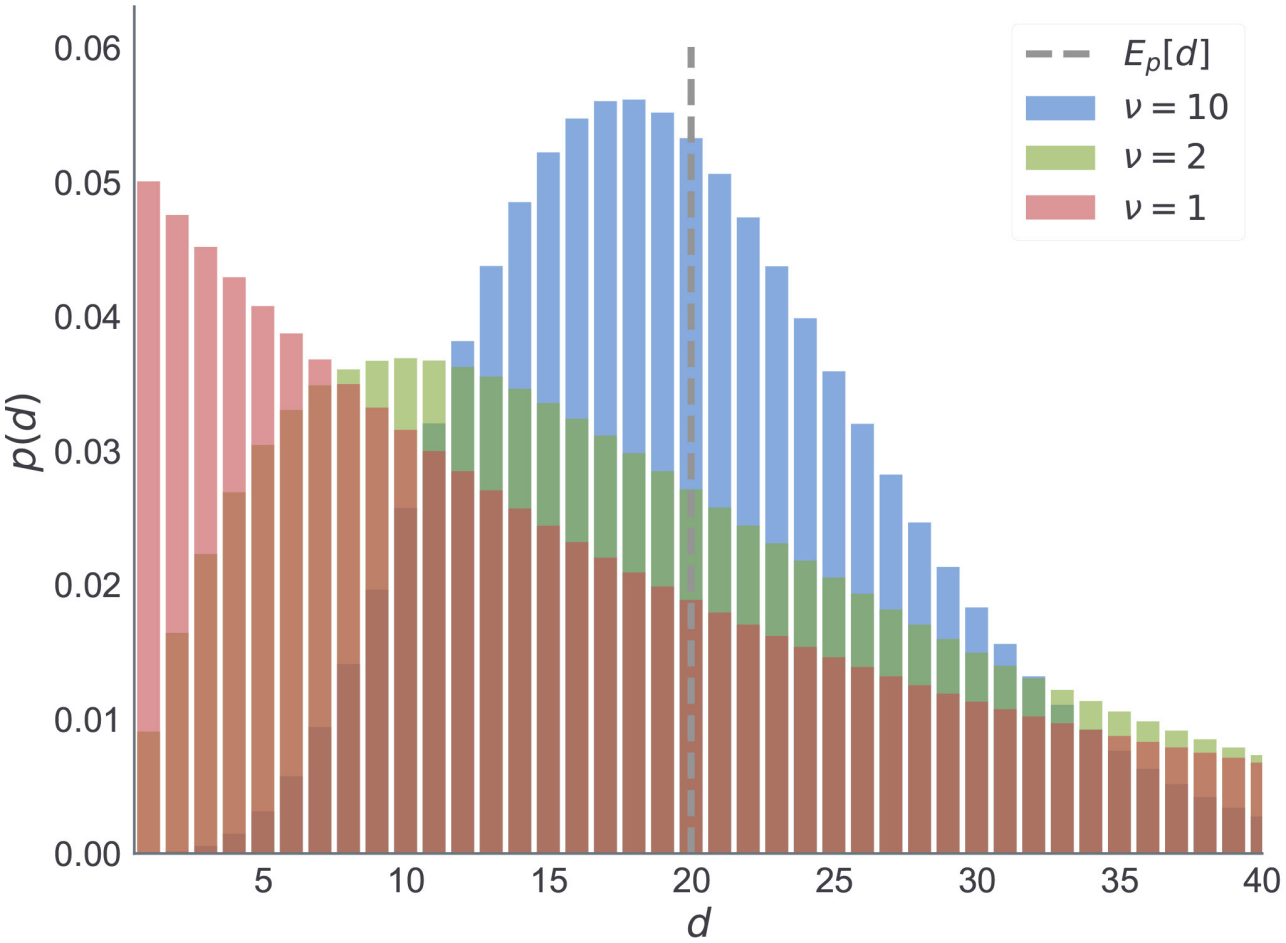
Negative binomial distribution. We illustrate here the changes in the negative binomial distribution as a function of shape parameter ν which is inversely proportional to the variance of between reversal durations. Note that for higher values of ν the distribution peaks around its expected value (dashed line). As the variance increases (green) the mode shifts toward zero. The limiting case of the negative binomial distribution in the form of geometric distribution (red) corresponds to ν = 1. For all three cases we fixed the mean duration to the same value.

Note that the parameter ν of the negative binomial distribution, acts as a precision parameter. We illustrate this in Figure 12.

The choice of prior beliefs about the between-reversal interval *d* in the form of a negative binomial distribution has interesting consequences on the dynamics of the marginal probability that a reversal will occur at some future point τ


(11) $$\begin{array}{l} \begin{array}{c} \delta_{m}\left[\tau \right]=p\left(s_{t+\tau }^{1}=2|s_{t-1}^{1}=2,f_{t-1}=\nu +1,m\right)\\ \;=\underset{f_{t},\ldots ,f_{t+\tau }}{\sum }\underset{s_{t}^{1},\ldots ,s_{t+\tau -1}^{1}}{\sum }p\left(s_{t}^{1},f_{t}|s_{t-1}^{1}=2,f_{t-1}=\nu +1,m\right)\\ \;\overset{t+\tau }{\underset{k=t+1}{\prod }}p\left(s_{k}^{1},f_{k}|s_{k-1}^{1},f_{k-1},m\right) \end{array} \end{array}$$

**Figure 13:**
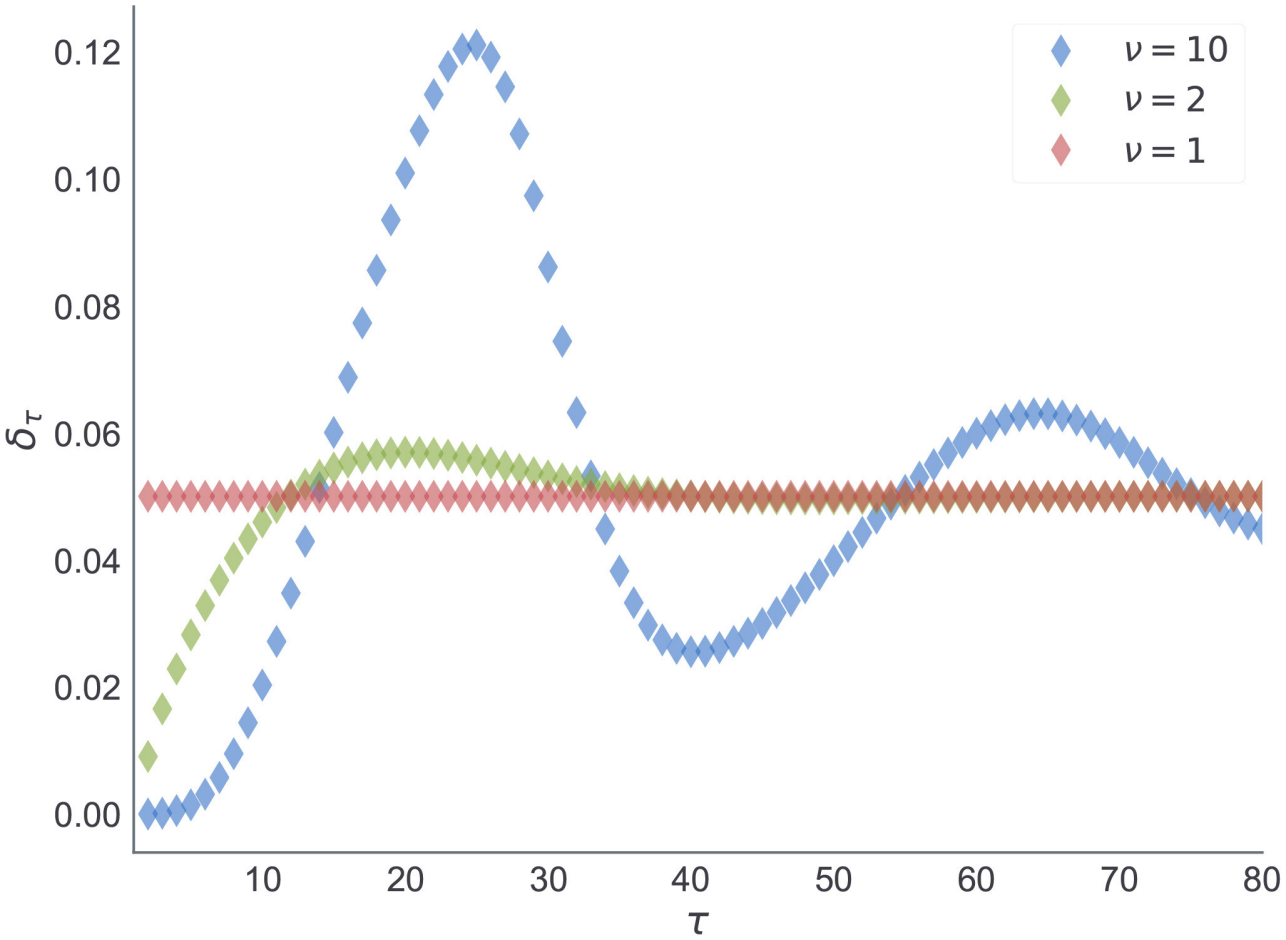
Expected transition probability at future trial τ. Estimate of the transition probability δ_*m*_[τ] (Equation 11), at a future trial τ conditioned upon a reversal at *t* and known initial state $s_{t}^{1}$. Each curve corresponds to estimates of the transition probability obtained from prior beliefs *p*_*m*_(*d*) shown in Figure 12.

In Figure 13, we show the dependence of the future reversal probability δ_*m*_[τ] on the precision parameter ν, given a fixed mean duration *E*_*p*_*m*__[*d*] = 20. Note that for ν = 1 we get a constant transition probability, which corresponds to the expectations of change probabilities found in hidden Markov models. In contrast, for larger values of ν one obtains a trial-dependent, effective transition probability with values alternating between low and high probabilities in a periodic manner. This temporal dependence of the transition probability will affect the inference process. The agent will become insensitive to subsequent reversals occurring a few trials after the previous reversal, and highly sensitive to reversals occurring twenty to thirty trials after the previous reversal.

Finally, the choice states $s_{t}^{2}$ are fully dependent on the current choice *a*_*t*_ ∈ {1, 2, 3}, and we express the state transition probability as


(12) $$\begin{array}{l} p\left(s_{t}^{2}|s_{t-1}^{2},a_{t}\right)=p\left(s_{t}^{2}|a_{t}\right)=\delta_{s_{t}^{2},a_{t}}\;. \end{array}$$

In practice this means that the agent is always certain about the choice it made and how that choice impacted the state of the task. Therefore, the posterior estimate over $s_{t}^{2}$ can be trivially expressed as


$$q\left(s_{t}^{2}|a_{t}\right)=\delta_{s_{t}^{2},a_{t}}\;.$$

#### 4.4.3. Active inference

In active inference, agents form posterior beliefs both about latent states of the environment and about their own actions. In other words, both perception and action selection are cast as inference problems (Attias, 2003; Botvinick and Toussaint, 2012). Practically, we will use variational inference for defining update rules for beliefs (Blei et al., 2017; Friston et al., 2017). In what follows we will first introduce perception as minimization of the variational free energy (upper bound on log-marginal likelihood) with respect to posterior beliefs over latent states, and after that introduce action selection as minimization of the expected free energy (Smith et al., 2022), that is, expected surprisal about future outcomes.

We write the generative model of outcomes *o*_*t*_ on trial *t* as


(13) $$\begin{array}{l} \tilde{p}(o_{t},s_{t}^{1},s_{t}^{2},f_{t},m,\rho )=p\left(o_{t}|s_{t}^{1},s_{t}^{2},\rho \right){\tilde{p}}_{t}\left(s_{t}^{1}|f_{t}\right)p\left(s_{t}^{2}|a_{t}\right)\\ {\tilde{p}}_{t}\left(f_{t}|m\right){\tilde{p}}_{t}(m){\tilde{p}}_{t}(\rho ), \end{array}$$

where we use ${\tilde{p}}_{t}\left(\cdot \right)$ to denote prior beliefs conditioned on a sequence of past outcomes, *o*_1:*t*−1_ = (*o*_1_, …, *o*_*t*−1_) and choices *a*_1:*t*−1_ = (*a*_1_, …, *a*_*t*−1_). Given a choice $a_{t}^{*}$ and an observed outcome *o*_*t*_ at trial *t*, the approximate posterior belief *q*_*t*_(*x*) over latent states $x=\left(s_{t}^{1},s_{t}^{2},f_{t},\rho ,m\right)$ is obtained in two steps:

- We first compute the marginal likelihood with respect to ${\tilde{p}}_{t}(\rho )$, and obtain the exact marginal posterior over discrete states using the Bayes rule
  (14) $$\begin{array}{l} q_{t}\left(s_{t}^{1},s_{t}^{2},f_{t},m\right)=\frac{{\tilde{p}}_{t}\left(o_{t},s_{t}^{1},s_{t}^{2},f_{t},m\right)}{{\tilde{p}}_{t}(o_{t})}. \end{array}$$
- Given the marginal posterior $q_{t}\left(s_{t}^{1},s_{t}^{2}\right)=\sum_{f_{t},m}q_{t}\left(s_{t}^{1},s_{t}^{2},f_{t},m\right)$ we compute the posterior over outcome probabilities using the variational message passing update
  (15) $$\begin{array}{l} q_{t}\left(\rho \right)\propto {\tilde{p}}_{t}\left(\rho \right)e^{\sum_{s_{t}^{1},s_{t}^{2}}q\left(s_{t}^{1},s_{t}^{2}\right)\ln\;p\left(o_{t}|s_{t}^{1},s_{t}^{2},\rho \right)}. \end{array}$$

As we initially defined the prior over outcome probabilities in the form of a Dirichlet distribution with parameters ***α***^**0**^, we can express the posterior estimate on every trial in the same functional form. Hence,


(16) $$\begin{array}{l} q_{t}\left(\rho \right)=\underset{s^{1}}{\prod }\underset{s^{2}}{\prod }Dir\left(\rho_{s^{1},s^{2}}|\alpha_{s^{1},s^{2}}^{t}\right) \end{array}$$

where


(17) $$\begin{array}{l} \begin{array}{c} \alpha_{s^{1},s^{2}=a_{t}^{*},i}^{t}=\delta_{o_{t},i}\cdot q\left(s_{t}^{1}=s^{1}\right)+\alpha_{s^{1},s^{2}=a_{t}^{*},i}^{t-1}\\ \alpha_{s^{1},s^{2}\neq a_{t}^{*},i}^{t}=\alpha_{s^{1},s^{2}\neq a_{t}^{*},i}^{t-1} \end{array}, \end{array}$$

and ${\tilde{p}}_{t}(\rho )=q_{t-1}(\rho )$. The above belief updating scheme corresponds to the variational surprise minimization learning algorithm (Liakoni et al., 2021; Markovic et al., 2021) adapted to the categorical likelihood and the Dirichlet prior.

#### 4.4.4. Action selection

In active inference, decision strategies (behavioral policies) are chosen based on a single optimization principle: minimizing expected surprisal about observed and future outcomes, that is, the expected free energy (Schwartenbeck et al., 2019; Smith et al., 2022). Here, we will express the expected free energy of a choice *a* on trial *t* as


(18) $$\begin{array}{l} G_{a}=\underset{\text{Risk}}{\underset{︸}{D_{KL}\left({\tilde{p}}_{t}(o_{t}|a)||P(o_{t})\right)}}+\underset{\text{Ambiguity}}{\underset{︸}{E_{{\tilde{p}}_{t}\left(s_{t}^{1}\right){\tilde{p}}_{t}\left(\rho \right)}\left[H\left[o_{t}|\rho ,s_{t}^{1},s_{t}^{2}=a\right]\right]}}\\ \approx -\underset{\text{Extrinsic value}}{\underset{︸}{E_{{\tilde{p}}_{t}(o_{t}|a)}\left[\ln P(o_{t})\right]}}\\ \;-\underset{\text{Epistemic value}}{\underset{︸}{E_{{\tilde{p}}_{t}(o_{t}|a)}\left[D_{KL}\left(q_{t}\left(s_{t}^{1},s_{t}^{2},f_{t}|o_{t},a\right)||{\tilde{p}}_{t}\left(s_{t}^{1},s_{t}^{2},f_{t}|a\right)\right)\right]}}\;\\ \;-\underset{\text{Novelty}}{\underset{︸}{E_{{\tilde{p}}_{t}(o_{t}|a)}\left[D_{KL}\left(q_{t}\left(\rho |o_{t},a\right)||{\tilde{p}}_{t}\left(\rho \right)\right)+D_{KL}\left(q_{t}\left(m|o_{t}\right),a||{\tilde{p}}_{t}\left(m\right)\right)\right]}} \end{array}$$

where *P*(*o*_*t*_) denotes prior preferences over outcomes, $H\left[o_{t}|\rho ,s_{t}^{1},s_{t}^{2}\right]$ the entropy of outcome likelihood $p\left(o_{t}|\rho ,s_{t}^{1},s_{t}^{2}\right)$, and *D*_*KL*_(*p*||*q*), stands for the Kullback-Leibler divergence between two probability densities : *p* and *q*. Note that action selection based on minimization of expected free energy would have an implicit dual imperative (see the different factorizations in Equation 18): On one hand, the expected free energy combines ambiguity and risk. On the other hand, it consists of information gain (epistemic value + novelty) and extrinsic value. Therefore, selecting actions that minimize the expected free energy dissolves the exploration-exploitation trade-off, as every action contains both expected value and information gain. This is a critical feature of action selection which allows us to account for epistemic choices as used in our experimental paradigm (see Figure 1).

At any trial *t* choice *a*_*t*_ is sampled from choice beliefs *p*(*a*_*t*_) (cf. planning as inference Attias, 2003; Botvinick and Toussaint, 2012) defined as


(19) $$\begin{array}{l} a_{t}\sim p\left(a_{t}|\gamma ,P_{o},\nu_{max}\right)\propto e^{-\gamma G_{a}[P_{o},\nu_{max},t]}, \end{array}$$

where parameter γ corresponds to choice precision, which we will attribute to empirical choice behavior of participants. Therefore, for describing participants' behavior we assume that the action selection process is corrupted by external sources of noise; e.g., mental processes irrelevant for the task at hand. In our simulations we will fix γ to a reasonably large value, to achieve approximate free energy minimization as the following relation will be satisfied


(20) $$\begin{array}{l} a_{t}\approx argmin_{a}G_{a},\text{ when }\gamma \gg 1. \end{array}$$

Notably, here we consider the simplest form of active inference in which expected free energy is computed from a one-step-ahead prediction. This is a standard simplification for environments in which actions cannot interfere with the state transitions, as is the case in typical dynamic multi-armed bandit problems (Markovic et al., 2021).

To express the expected free energy, *G*(*a*_*t*_), in terms of beliefs about arm-specific reward probabilities, we will first constrain the prior preference to the following categorical distribution


(21) $$P(o_{t})=\prod_{o_{t}}\;{\left[P_{o}\right]}^{\delta_{o,o_{t}}},\;P_{o}=\left(p_{-},p_{+},\frac{1}{2}p_{c},\frac{1}{2}p_{c}\right)$$

In active inference, prior preferences determine whether a particular outcome is attractive, that is, rewarding. Here we assume that all agents prefer gains (*o*_*t*_ = 2) over losses (*o*_*t*_ = 1). Hence, we constrain parameter values such that *p*_+_ > *p*_−_ holds always. The ratio $\frac{p_{+}}{p_{c}}=\lambda$ determines the balance between epistemic and pragmatic imperatives. When prior preferences for gains are very precise, corresponding to large λ, the agent becomes risk sensitive and will tend to forgo exploration if the risk is high (see Equation 18). Conversely, a low lambda corresponds to an agent which is less sensitive to risk and will engage in exploratory, epistemic behavior, until it has familiarized itself with the environment.

Given the following expressions for the marginal predictive likelihood,


(22) $$\begin{array}{l} {\tilde{p}}_{t}\left(o_{t}|a\right)=\sum_{s_{t}^{1},s_{t}^{2}}\int p\left(o_{t}|\rho ,s_{t}^{1},s_{t}^{2}\right){\tilde{p}}_{t}\left(s_{t}^{1}\right)p\left(s_{t}^{2}|a\right){\tilde{p}}_{t}\left(\rho \right)\text{ d}\rho \\ {\tilde{p}}_{t}\left(o_{t}|a\right)=\sum_{s=1}^{2}{\tilde{p}}_{t}\left(s_{t}^{1}=s\right)\prod_{o=1}^{4}{\left[\mu_{s,a,o}^{t-1}\right]}^{\delta_{o_{t},o}}\\ \mu_{s^{1},s^{2},o}^{t-1}=\frac{\alpha_{s^{1},s^{2},o}^{t-1}}{\sum_{i}\alpha_{s^{1},s^{2},i}^{t-1}},\;{\bar{\mu }}_{s^{2},o}^{t-1}=\sum_{s^{1}}{\tilde{p}}_{t}\left(s_{t}^{1}=s^{1}\right)\mu_{s^{1},s^{2},o}^{t-1} \end{array}$$

we get the following expressions for the expected free energy


(23) $$\begin{array}{l} \begin{array}{c} G_{t}\left(a\right)=\underset{o}{\sum }{\bar{\mu }}_{a,o}^{t-1}\ln\;\frac{{\bar{\mu }}_{a,o}^{t-1}}{P_{o}}-\underset{s^{1}}{\sum }\tilde{p}\left(s_{t}^{1}=s^{1}\right)\\ \underset{o}{\sum }\mu_{s^{1},a,o}^{t-1}\left(\psi \left(\alpha_{s^{1},a,o}^{t-1}+1\right)-\psi \left(1+\underset{j}{\sum }\alpha_{s^{1},a,j}^{t-1}\right)\right) \end{array} \end{array}$$

Above we have used the following relation


(24) $$\begin{array}{l} \int \text{d}xDir\left(x|\alpha \right)x_{i}\ln x_{i}=\\ \;\frac{\alpha_{i}}{\sum_{j}\alpha_{j}}\left(\psi \left(\alpha_{i}+1\right)-\psi \left(1+\sum_{j}\alpha_{j}\right)\right), \end{array}$$

for computing ambiguity term in Equation (18).

### 4.5. Model inversion

To estimate subject-specific priors we effectively identified prior beliefs (i.e., ν_*max*_, γ, and ***P***_*o*_) that rendered the observed choices the most likely under active inference (i.e., under ideal Bayesian assumptions and the complete class theorem). In other words, for any given (ν_*max*_, γ, ***P***_*o*_), we can simulate belief updating — given subject specific outcomes to evaluate the expected free energy. The expected free energy then specifies the probability of choices at each trial. These probabilities can be used to assess the likelihood of any observed choice sequence of *n*th subject, conditioned upon a particular set of priors [*p*(ν_*max*_, γ, ***P***_*o*_)]. One can then explore the space of priors (i.e., model parameters) to evaluate the marginal likelihood or model evidence for different combinations of priors.

In more detail, given a sequence of subjects' responses $A^{n}=\left(a_{1}^{n},\ldots ,a_{T}^{n}\right)$, where *n* denotes subject index and *T* = 1, 000 denotes the total number of trials, the response likelihood is defined as


(25) $$\begin{array}{l} P\left(A^{n}|\gamma ,P_{o},\nu_{max}\right)=\overset{T}{\underset{t=400}{\prod }}p\left(a_{t}=a_{t}^{n}|\gamma^{n},P_{o}^{n},\nu_{max}^{n}\right). \end{array}$$

Note that for estimating the posterior over model parameters (γ, ***P***_*o*_, ν_*max*_) we ignore the first 400 responses from the likelihood. We expect that during these first trials, subjects are still getting used to the task, and potentially use additional strategies for representing the task and making choices. As we do not model all possible task representations, exclusion of initial trails reduces the noise in model comparison. Importantly, we do use the entire set of responses for computing belief trajectories of the active inference agents, that is, we expose the agent to the complete sequence of individual responses and the corresponding outcomes.

We define the prior over model parameters $\left(\nu_{max}^{n},\gamma^{n},P_{o}^{n}\right)$ for the *n*th subject as follows:


(26) $$\begin{array}{l} p\left(\gamma^{n},P_{o}^{n},\nu_{max}^{n}\right)=p(\gamma^{n})p(P_{o})p\left(\nu_{max}^{n}\right), \end{array}$$

where for a prior over choice precision parameter γ we use an inverse gamma distribution, thus


(27) $$\begin{array}{l} p(\gamma^{n})\sim \Gamma^{-1}(2,2), \end{array}$$

and for the prior over prior preferences ***P***_*o*_ we use a Dirichlet distribution, such that


(28) $$\begin{array}{l} \begin{array}{c} p_{i}^{n}\sim Dir(p|\beta ),\;\beta_{i}=1,i\in \{1,2,3\},\\ P_{o}^{n}=\left(\frac{p_{1}^{n}}{2},\frac{p_{1}^{n}}{2}+p_{2}^{n},\frac{p_{3}^{n}}{2},\frac{p_{3}^{n}}{2}\right). \end{array} \end{array}$$

With the above parameterization of prior preferences ***P***_*o*_ we constrain the prior to reflect our expectations that all subjects will have higher preferences for gains than for losses, and that they will have equivalent preference associated with informative cues, that is, epistemic choices. Finally, we define a prior over the temporal precision parameter ν_*max*_ as a categorical distribution


(29) $$\begin{array}{l} \nu_{max}^{n}\sim Cat\left(r^{n}\right) \end{array}$$

where $r^{n}=\left(r_{1}^{n},\ldots ,r_{10}^{n}\right)$ denotes prior probability over possible ν_*max*_ values. Here we adopt the approach known as random effect Bayesian model selection (Stephan et al., 2009; Rigoux et al., 2014) which treats models (i.e., different ν_*max*_ values) as random effects that could differ between subjects and conditions, with an unknown population distribution. Hence, we introduce a condition specific hyper-priors over model probabilities in the form of a Dirichlet distribution


(30) $$\begin{array}{l} \begin{array}{c} \;\tau \sim C^{+}\left(0,1\right)\\ r_{1}\sim Dir(r_{1}|\alpha_{0}/\tau )\\ r_{2}\sim Dir(r_{2}|\alpha_{0}/\tau ) \end{array} \end{array}$$

where ***r***_1_ corresponds to the condition with regular reversals and ***r***_2_ to the condition with the irregular reversals. Finally, τ plays a role of a shrinkage parameter, that sets a non-zero probability to a configuration where all models have equal frequency in the population (in the limit τ → 0 we get $r_{1}=r_{2}=\frac{1}{10}$). The subject specific prior probability ***r***^*n*^ corresponds to one of the two priors, based on the condition the subject was exposed to; hence, $r^{n}\in \{r_{1},\;r_{2}\}$.

To implement the above hierarchical generative model of subjects responses we used the probabilistic programming library Numpyro (Phan et al., 2019). Numpyro library provides an interface to multiple state-of-the-art inference schemes. For drawing samples from the posterior we have used Numpyro's implementation of the No-U-Turn sampler (NUTS) (Hoffman et al., 2014). NUTS is an self-tuning version of the Hamiltonian Monte Carlo, a popular Markov Chain Monte Carlo algorithm for avoiding random walks and sensitivity to between-parameter correlations. The limitation of NUTS is that it can only draw samples from continues random variables. Therefore, for implementation purposes we have to marginalize the generative model with respect to ν_*max*_.

The marginalization results in the following marginal generative model:


(31) $$\begin{array}{l} \begin{array}{c} \tau \sim C^{+}\left(0,1\right)\\ r_{1}\sim Dir\left(r_{1}|\alpha_{0}/\tau \right),\;\alpha_{0,\nu }=1,\nu \in \{1,\ldots ,10\},\\ r_{2}\sim Dir\left(r_{2}|\alpha_{0}/\tau \right),\;\alpha_{0,\nu }=1,\nu \in \{1,\ldots ,10\},\\ r^{n}=f\left(r_{1},r_{2},n\right)\\ P_{o}^{n}\sim p\left(P_{o}^{n}|\beta \right),\;\beta_{i}=1,i\in \{1,2,3\},\\ \gamma^{n}\sim \Gamma^{-1}\left(2,2\right),\\ A^{n}\sim \underset{\nu }{\sum }r_{\nu }^{n}\overset{1000}{\underset{t=400}{\prod }}p\left(a_{t}|\gamma^{n},P_{o}^{n},\nu_{max}=\nu \right). \end{array} \end{array}$$

With the mixture model above we can unify the parameter estimation with the model comparison (selection). Given a sample from the posterior


(32) $$\begin{array}{l} r_{1}^{s},r_{2}^{s},P_{o}^{n,s},\gamma^{n,s}\sim p\left(r_{1},r_{2},P_{o}^{1:N},\gamma^{1:N}|A^{1:N}\right) \end{array}$$

we can obtain a sample from the marginal posterior probability over ν_*max*_ for the *n*th subject as


(33) $$\begin{array}{l} p^{s}\left(\nu_{max}^{n}=\nu |A^{1:N}\right)=\frac{p\left(A^{n}|\gamma^{n,s},P_{o}^{n,s},\nu_{max}^{n}=\nu \right)r_{\nu }^{n,s}}{\sum_{i}p\left(A^{n}|\gamma^{n,s},P_{o}^{n,s},\nu_{max}^{n}=i\right)r_{i}^{n,s}}\;. \end{array}$$

To classify subjects' behavior in terms of adaptability of temporal representations we use the exceedance probability (Rigoux et al., 2014) of the marginal posterior defined as


(34) $$\begin{array}{l} \begin{array}{c} i^{n,s}=argmax_{\nu }p^{s}\left(\nu_{max}^{n}=\nu |A^{1:N}\right),\\ X_{i}^{n}=\frac{1}{S}\overset{S}{\underset{s=1}{\sum }}\delta_{i,i^{n,s}}, \end{array} \end{array}$$

thus, obtaining the probability that the *i*th model has the highest marginal posterior probability for the *n*th subject. The value $X_{i}^{n}$ is plotted in Figure 8. Finally, the most likely precision parameter $\nu_{max}^{n}$ of the *n*th subject corresponds to $\nu_{max}^{n}=\;argmax_{i}X_{i}^{n}$ which we than used for classification as illustrated in Figures 9, 10.

## Data availability statement

The datasets presented in this study can be found in online repositories. The names of the repository/repositories and accession number(s) can be found at: https://osf.io/h526v/.

## Ethics statement

The studies involving human participants were reviewed and approved by the Ethical Board of Technical University Dresden. The patients/participants provided their written informed consent to participate in this study.

## Author contributions

DM, AR, and SK contributed to conception and design of the study and wrote the sections of the manuscript. DM and AR collected the data. DM developed the model, performed the data analysis, and wrote the first draft of the manuscript. All authors contributed to manuscript revision, read, and approved the submitted version.

## Funding

The study was supported by the German Research Foundation (DFG, Deutsche Forschungsgemeinschaft), SFB 940/3—Project A09 awarded to SK and SFB 940/3—Project B7 awarded to AR. SK acknowledges further support by DFG TRR 265/1 (Project ID 402170461, B09) and Germany's Excellence Strategy—EXC 2050/1 (Project ID 390696704)—Cluster of Excellence Centre for Tactile Internet with Human-in-the-Loop (CeTI) of Technische Universität Dresden. AR acknowledges further support by the German Research Foundation (DFG RE 4449/1-1, RTG 2660-B2) and by a 2020 BBRF NAR-SAD Young Investigator Grant from the Brain and Behavior Research Foundation. This study was partially funded by funding opportunities for young scientists (Anschubfinanzierung) from the Department of Psychology of the Technische Universität Dresden.

## Conflict of interest

The authors declare that the research was conducted in the absence of any commercial or financial relationships that could be construed as a potential conflict of interest.

## Publisher's note

All claims expressed in this article are solely those of the authors and do not necessarily represent those of their affiliated organizations, or those of the publisher, the editors and the reviewers. Any product that may be evaluated in this article, or claim that may be made by its manufacturer, is not guaranteed or endorsed by the publisher.
