# Supplementary material for: Revealing human sensitivity to a latent temporal structure of changes
Source: Front Behav Neurosci. 2022 Oct 17;16:962494. doi: 10.3389/fnbeh.2022.962494 (PMC9621332; doi:10.3389/fnbeh.2022.962494)
Supplement: Supplementary file 1 [file Data_Sheet_1.pdf]

Supplementary material

1 SUPPLEMENTARY FIGURES

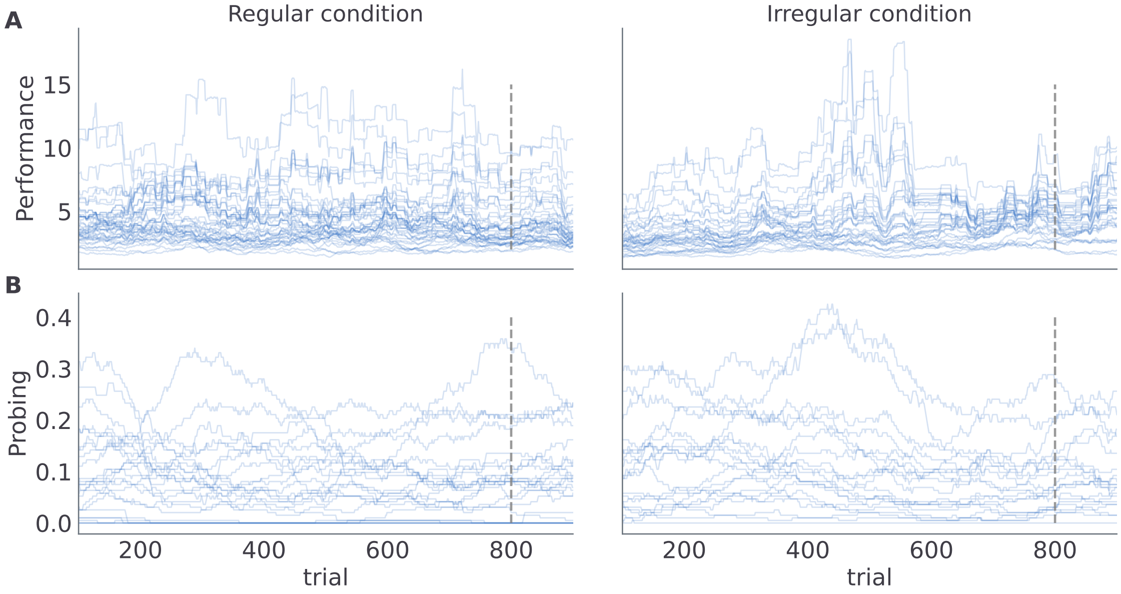

Figure S1. Subject specific trajectories of behavioural measures. Each line corresponds to a trajectory of: **A** performance, and **B** probing for individual subjects.

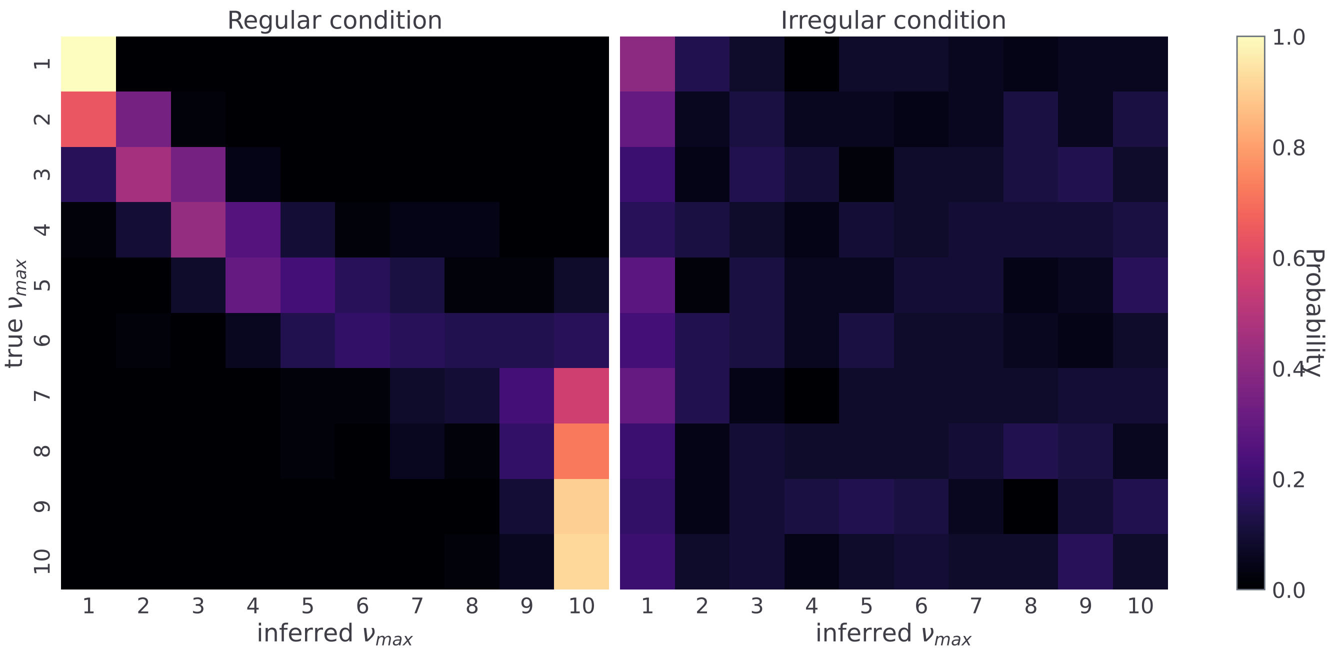

Figure S2. Confusion matrix estimated using simulated data. Probability of assigning simulated behavioural responses to possible ν_{max} values given the true $\nu_{max} \in \{1, \dots, 10\}$. The confusion matrix was estimated based on $n = 100$ simulated responses (50 simulated subjects in each condition) for each generative ν_{max} value. In all simulation the remaining free model parameters were fixed to $\gamma = 5$, and $\boldsymbol{P}_o = (0.1, 0.6, 0.15, 0.15)$.

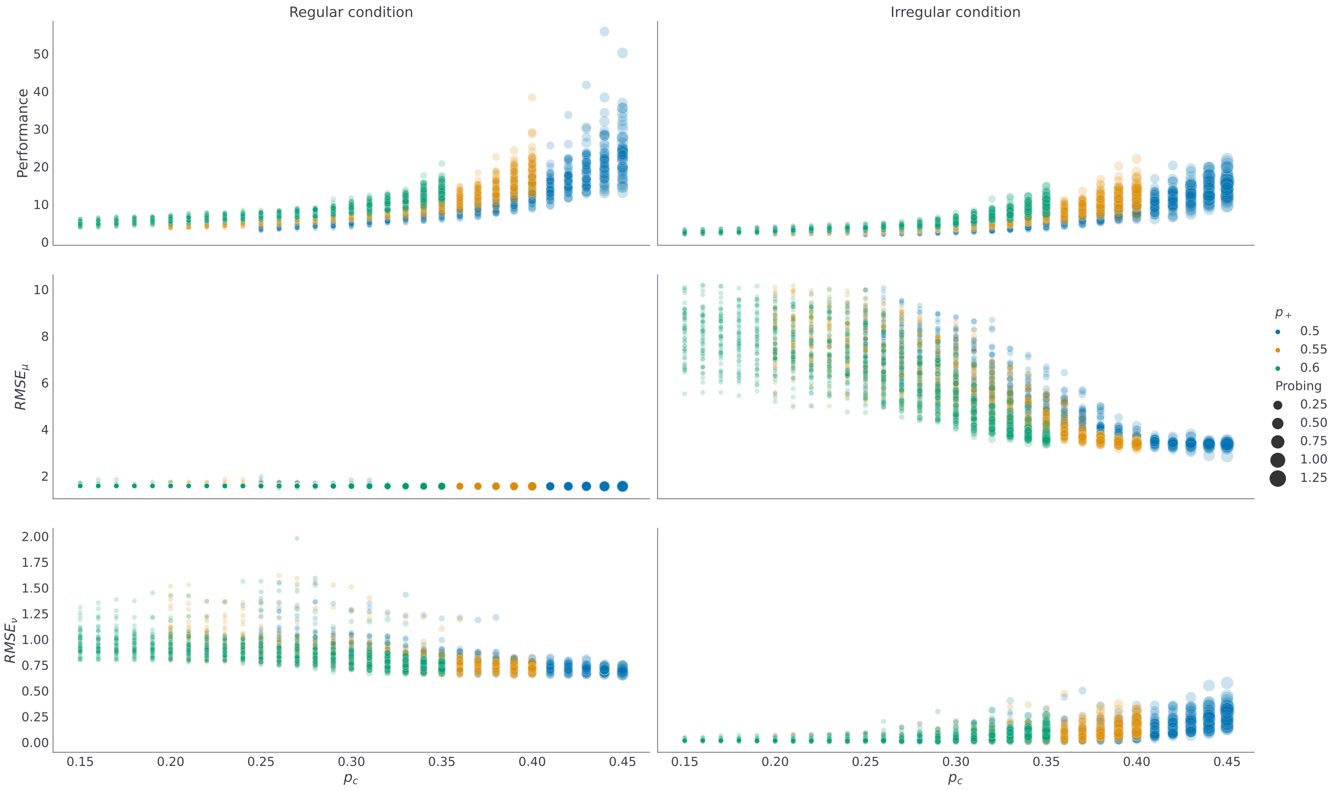

Figure S3. Dependence of performance, probing, and the quality of temporal representations on prior preference over cues p_c. As in the main figure each line corresponds to an average over $n = 50$ simulated trajectories with $\nu_{max} = 10$ and $\gamma = 5$.
